# NMR analysis of the interaction of picornaviral proteinases Lb and 2A with their substrate eukaryotic initiation factor 4GII

**DOI:** 10.1002/pro.2807

**Published:** 2015-10-04

**Authors:** Martina Aumayr, Sofiya Fedosyuk, Katharina Ruzicska, Carla Sousa‐Blin, Georg Kontaxis, Tim Skern

**Affiliations:** ^1^ Department of Medical BiochemistryMax F. Perutz Laboratories, Medical University of Vienna, Vienna BiocenterDr. Bohr‐Gasse 9/3ViennaA‐1030Austria; ^2^ Department of Structural and Computational BiologyMax F. Perutz Laboratories, University of ViennaCampus Vienna Biocenter 5ViennaA‐1030Austria; ^3^ Carla Sousa‐Blin's current address is MRC Centre for Regenerative Medicine, SCRM Building, The University of Edinburgh bioQuarter5 Little France DriveEdinburghEH16 4UUUnited Kingdom

**Keywords:** viral proteases, substrate binding, virus–host interactions, protein–protein interactions, control of protein synthesis

## Abstract

Messenger RNA is recruited to the eukaryotic ribosome by a complex including the eukaryotic initiation factor (eIF) 4E (the cap‐binding protein), the scaffold protein eIF4G and the RNA helicase eIF4A. To shut‐off host–cell protein synthesis, eIF4G is cleaved during picornaviral infection by a virally encoded proteinase; the structural basis of this reaction and its stimulation by eIF4E is unclear. We have structurally and biochemically investigated the interaction of purified foot‐and‐mouth disease virus (FMDV) leader proteinase (Lb^pro^), human rhinovirus 2 (HRV2) 2A proteinase (2A^pro^) and coxsackievirus B4 (CVB4) 2A^pro^ with purified eIF4GII, eIF4E and the eIF4GII/eIF4E complex. Using nuclear magnetic resonance (NMR), we completed ^13^C/^15^N sequential backbone assignment of human eIF4GII residues 551–745 and examined their binding to murine eIF4E. eIF4GII_551–745_ is intrinsically unstructured and remains so when bound to eIF4E. NMR and biophysical techniques for determining stoichiometry and binding constants revealed that the papain‐like Lb^pro^ only forms a stable complex with eIF4GII_551–745_ in the presence of eIF4E, with *K*_D_ values in the low nanomolar range; Lb^pro^ contacts both eIF4GII and eIF4E. Furthermore, the unrelated chymotrypsin‐like 2A^pro^ from HRV2 and CVB4 also build a stable complex with eIF4GII/eIF4E, but with *K*_D_ values in the low micromolar range. The HRV2 enzyme also forms a stable complex with eIF4E; however, none of the proteinases tested complex stably with eIF4GII alone. Thus, these three picornaviral proteinases have independently evolved to establish distinct triangular heterotrimeric protein complexes that may actively target ribosomes involved in mRNA recruitment to ensure efficient host cell shut‐off.

**Abbreviations**
CTE: C‐terminal extension
CV: coxsackievirus
eIF: eukaryotic initiation factor
FMDV: foot‐and‐mouth disease virus
HRV: human rhinovirus 2
HSQC: heteronuclear single quantum coherence
IDP: intrinsically disordered protein
IRES: internal ribosome entry site
ITC: isothermal titration calorimetry
MALLS: multi‐angle laser light scattering
NMR: nuclear magnetic resonance
PABP: polyA binding protein
PRE: paramagnetic relaxation enhancement
PV: poliovirus
RRL: rabbit reticulocyte lysate
SEC: size‐exclusion chromatography

## Introduction

**Figure 1:**
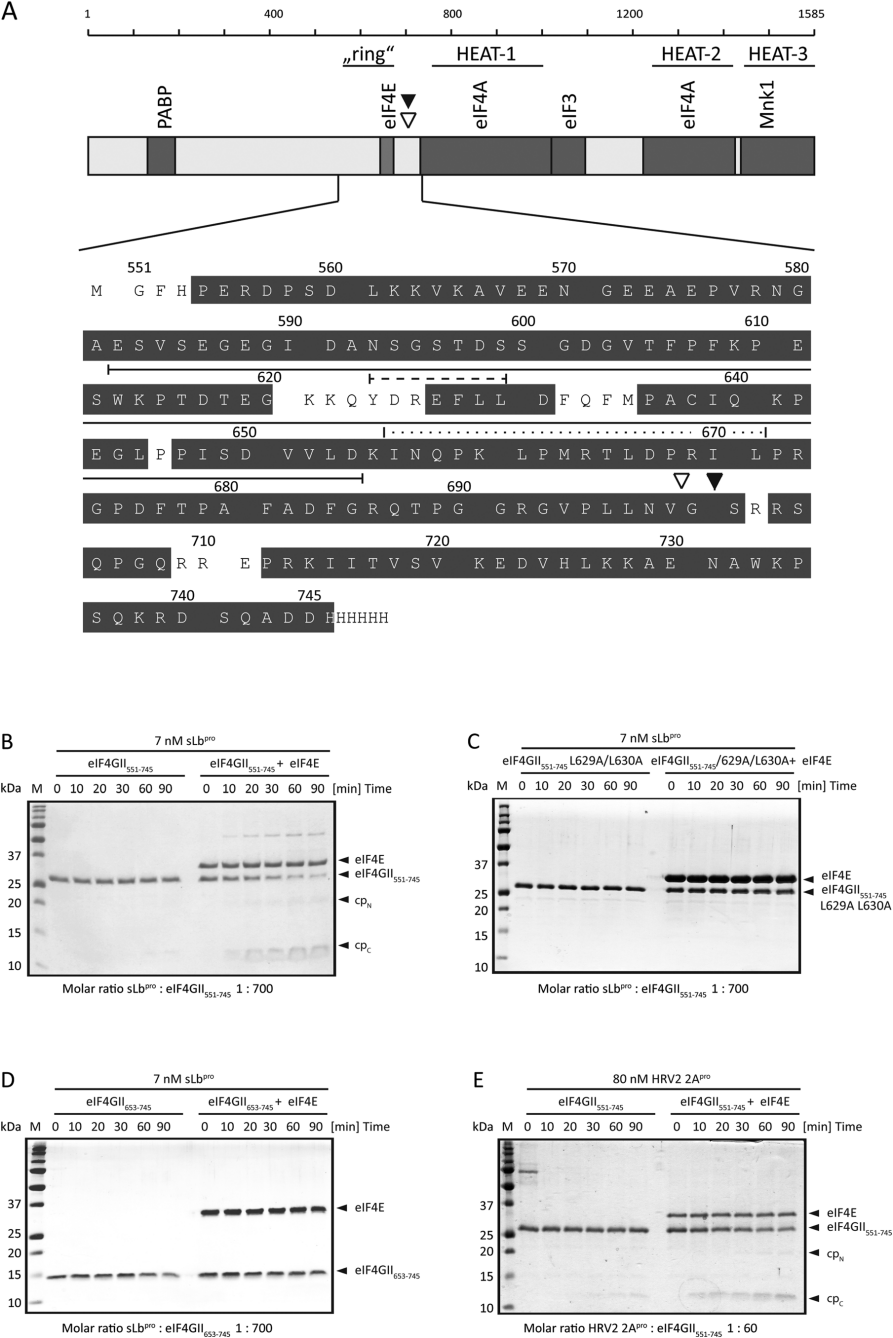
Domain structure of human eIF4GII and cleavage of eIF4GII_551–745_ by picornaviral proteases. (A) Schematic representation of human eIF4G with known structural domains indicated (the “ring” domain has only been shown in yeast) and the amino acid sequence of the fragment of eIF4GII_551–745_ used. Protein binding sites are indicated as follows: eIF4E binding site, broken line; Lb^pro^ binding site, dotted line; 2A^pro^ binding region, straight line. Positions of the cleavage site of the Lb^pro^ and 2A^pro^ are marked by black and white triangles, respectively. The backbone NMR signals of 178 residues underlaid in black were assigned using triple resonance experiments, (B) *In vitro* cleavage of 5 µ*M* eIF4GII_551–745_ by 7 n*M* sLb^pro^ in the absence or the presence of 5 µ*M* eIF4E, (C) *In vitro* cleavage of 5 µ*M* eIF4GII_551–745_L629A/L630A by 7 n*M* of sLb^pro^ in the absence or the presence of 25 µ*M* eIF4E, (D) *In vitro* cleavage of 11 µ*M* eIF4GII_653‐745_ by 7 n*M* of sLb^pro^ in the absence or the presence of 5 µ*M* eIF4E, (E*) In vitro* cleavage of 5 µ*M* eIF4GII_551–745_ by 80 n*M* HRV2 2A^pro^ in the absence or the presence of 5 µ*M* eIF4E. The molar ratio of sLb^pro^/HRV2 2A^pro^ to eIF4GII_551–745_ is indicated below each cleavage figure.

Successful viral replication requires rapid modulation of the infected cell's physiology. Interference with the host cell's machinery for protein synthesis is a mechanism favoured by positive strand RNA viruses to increase the efficiency of viral mRNA translation. The shut‐off of host cell protein synthesis by picornaviruses, first observed in 1963 by Penman *et al*.,^1^ is a classic example. The effect has since been observed during replication of poliovirus (PV), coxsackievirus (CV) and human rhinovirus (HRV) (members of the enterovirus genus) and foot‐and‐mouth disease virus (FMDV, a member of the aphthovirus genus).^2^, ^3^ The shut‐off occurs one to two hours post‐infection and before replication of the RNA genome takes place; thus, the reaction is efficiently carried out by proteinases translated from the RNA genome of the infecting particle.^4^ Cleavage of the two isoforms of the cellular eukaryotic initiation factor (eIF) 4G, eIF4GI and eIF4GII [Fig. 1(A)], observed in all enteroviruses and aphthoviruses so far examined, separates the domain that binds the polyA binding protein (PABP) and eIF4E (the cap‐binding protein) from that binding eIF4A (an ATP‐dependent RNA helicase), the protein complex eIF3 that binds to the 40S ribosomal subunit and the growth factor sensitive Mnk‐1 kinase.^5^, ^6^ Consequently, the host cell fails to recruit capped mRNA to the ribosome; in contrast, viral RNA translation is initiated through the internal ribosome entry site (IRES) at the RNA's 5' end.^7^ In FMDV, the shut‐off of host cell translation through cleavage of eIF4G prevents synthesis of interferons and related cytokines, thus impairing host innate immunity.^8^

Cleavage of eIF4G isoforms in FMDV is performed by the Leader proteinase (Lb^pro^), a papain‐like cysteine proteinase, with a globular domain of 155 amino acids and a C‐terminal extension (CTE) of 18 amino acids. In enteroviruses, the cleavage is performed by the chymotrypsin‐like 2A proteinase (2A^pro^). The outcome of the cleavage of the two isoforms of eIF4G is the same; nevertheless, the cleavage sites and the mechanisms leading to the cleavage are not identical. Thus, on eIF4GI, the cleavages sites are located 7 amino acids apart whereas in eIF4GII, they are adjacent [Fig. 1(A)].^9^ Prior to cleavage of the eIF4G isoforms, Lb^pro^ binds to an exosite on the eIF4G isoforms located between 30 and 50 amino acids N‐terminal to the cleavage site [Fig. 1(A)]; Lb^pro^ residues required for this binding include C133 as well as 183–195 of the CTE. In contrast, the 2A^pro^ of HRV2 binds to an ill‐defined exosite region on eIF4G between 20 and 80 residues N‐terminal to the cleavage site [Fig. 1(A)]. The HRV2 2A^pro^ residues involved in binding include residues L17–Y32 but have not been fully explored. Exosite binding by both Lb^pro^ and HRV2 2A^pro^ in GST pull‐down experiments using full‐length eIF4GI from rabbit reticulocyte lysates (RRLs) requires the presence of eIF4E;^10^, ^11^ furthermore, cleavage of both full‐length and suitable fragments of eIF4G isoforms by both enzymes is stimulated in the presence of eIF4E in RRLs and in human 293 cells.^9^, ^12^, ^13^ Indeed, with the Lb^pro^, cleavage of full‐length eIF4GI in RRLs could be completely abrogated by addition of eIF4E‐binding protein to complex the eIF4E.^13^ The stoichiometry and structural basis of the stimulation of eIF4G isoform cleavage by eIF4E are unknown.

Structural information on the two proteinases as well as yeast and mammalian eIF4E is available.^14^, ^15^, ^16^, ^17^, ^18^ For mammalian eIF4G, structural information^19^, ^20^, ^21^ is limited to regions C‐terminal to the cleavage sites of the picornaviral proteinases [Fig. 1(A)]; nevertheless, the interaction of human eIF4E with human eIF4GI residues 557–646 has been characterised by isothermal titration calorimetry (ITC).^22^ For yeast, the solution structure of residues 393–490 bound to yeast eIF4E has been determined, with residues 393–490 forming a ring around the N‐terminus of eIF4E.^23^ However, the yeast eIF4G is not cleaved by the HRV2 2A^pro^ or the CVB4 2A^pro^ 24 and the sequence C‐terminal to the eIF4E binding site shows low similarity to that found in mammalian eIF4G, putting into question the relevance of this structure for this region of the mammalian protein. Supporting Information Figure S1 aligns the amino acid sequence of yeast eIF4G with those of human eIF4GI and eIF4GII (Supporting Information Fig. S1). The identity of the eIF4GII_551–745_ fragment to the corresponding regions of human eIF4GI and yeast eIF4GI is 48% and 20%, respectively.

To investigate the interaction of FMDV Lb^pro^ as well as HRV2 and CVB4 2A^pro^ with mammalian eIF4G and the eIF4G/eIF4E complex and the mechanism of stimulation of cleavage by eIF4E, we biochemically and biophysically characterised the formation of protein complexes between three picornaviral proteinases and the mammalian translation initiation factors and structurally characterised changes in eIF4GII and Lb^pro^ and HRV2 2A^pro^ by NMR. The eIF4GII fragment examined remained intrinsically unstructured even when bound to eIF4E. Lb^pro^ or 2A^pro^ from either HRV2 or CVB4 both form a ternary complex with eIF4GII and eIF4E; Lb^pro^ and HRV2 2A^pro^ recognise both eIF4G and eIF4E in their respective complexes. However, sLb^pro^, HRV2 2A^pro^ and CVB4 2A^pro^ interact differently with eIF4GII_551–745_ and eIF4E.

## Results

### eIF4GII_551–745_, eIF4E and sLb^pro^ form a tight heterotrimeric complex

To obtain structural information on the interaction of eIF4G with the picornaviral proteinases, we over‐expressed and purified a fragment comprising residues 551–745 (eIF4GII_551–745_) of the 1585 amino acid human eIF4GII protein. This fragment contains the eIF4E binding site^6^, ^25^ as well as the Lb^pro^ and HRV 2A^pro^ binding and cleavage sites [Fig. 1(A)]. To ensure that this fragment behaves as the corresponding region of the full‐length protein, we investigated its cleavage by purified recombinant picornaviral proteinases. For Lb^pro^, we used a naturally occurring shortened form (termed sLb^pro^) lacking six amino acids at the C‐terminus.^16^, ^26^ This deletion precludes protease dimerisation via interactions of the C‐terminus of one molecule with the substrate binding site of a second and vice‐versa,^27^, ^28^ thus simplifying complex formation and structural studies thereof.^29^ For the 2A^pro^, we used wild‐type HRV2 and CVB4 2A^pro^.

First, we wished to quantify the stimulation of picornaviral cleavage of eIF4GII_551–745_ using the purified proteins; no data on the exact extent of stimulation are available. Thus, in an activity assay, cleavage of 1 µg (5 µ*M*) of eIF4GII_551–745_ was faintly visible in the presence of 1 ng sLb^pro^ (concentration 0.125 µg/mL; 7 n*M*) following 90 min of incubation [Fig. 1(B)]. However, the presence of 1 µg (5 µ*M*) of recombinant full‐length murine eIF4E purified from supernatants of *E.coli* lysates, cleavage of eIF4GII_551–745_ was already detectable after 10 min and 50% cleavage was reached between 20 and 30 minutes [Fig. 1(B)]. N‐terminal sequencing and mutational analysis both confirmed that cleavage of sLb^pro^ on eIF4GII_551–745_ was between residues G700 and S701, as determined previously.^9^

To obtain cleavage of eIF4GII_551–745_ in the absence of eIF4E, incubation with 40 ng (260 n*M*) of sLb^pro^ for 60 minutes was required [Supporting Information Fig. S2(A)], whereas only 1 ng (7 n*M*) was required in the presence of eIF4E [Fig. 1(B)]. The N‐terminal cleavage product (cp_N_) was however always only poorly visible. Subsequent experiments using residues eIF4GII_551‐700_ (corresponding to the N‐terminal cleavage fragment) as substrate identified a further cleavage site for sLb^pro^ in the presence of eIF4E (data not shown). To eliminate the possibility that the simple presence of eIF4E was affecting sLb^pro^ cleavage, we constructed a version of eIF4GII_551–745_ bearing the mutations L629A and L630A (eIF4GII_551–745_L629A/L630A) in the eIF4E binding site. eIF4E failed both to form a complex with eIF4GII_551–745_L629A/L630A (data not shown) and to stimulate cleavage of the mutated eIF4GII fragment, even when eIF4E was added in excess [Fig. 1(C)]. Furthermore, we also examined the cleavage of the eIF4GII_653‐745_ fragment which lacks the binding site for eIF4E. No cleavage by sLb^pro^ at 7 n*M* in the presence or absence of eIF4E was observed [Fig. 1(D)]. When a 10‐fold molar excess of sLb^pro^ [Supporting Information Fig. S2(B)] was added, cleavage of this fragment could be observed; however, this level of cleavage was not increased by the addition of eIF4E [Fig. S2(B)]. Together, these experiments show that formation of the eIF4GII:eIF4E complex stimulates sLb^pro^ cleavage of eIF4GII_551–745_ between 4 and 40 fold. This level was independent of the presence or absence of 5 µ*M* of the cap analogue m7G(5')ppp(5')G (data not shown).

The eIF4GII_551–745_ fragment was also cleaved by HRV2 2A^pro^ [Fig. 1(E)] and CVB4 2A^pro^ (data not shown). However, 10 ng (80 n*M*) of enzyme were required in the absence of eIF4E to obtain detectable cleavage fragments after 60‐90 minutes. The presence of an equimolar amount of eIF4E also stimulated HRV2 and CVB4 2A^pro^ cleavage; however, the stimulation was less efficient than that seen with sLb^pro^, with 50% cleavage only being observed after 60 minutes [Fig. 1(E) and data not shown].

In contrast to the stimulation of picornaviral proteinase cleavage, the presence of eIF4E reduced unspecific digestion of the eIF4GII_551–745_ fragment by broad specificity proteinases, such as trypsin and elastase (Supporting Information Fig. S3), indicating that the stimulation of cleavage by eIF4E was a specific effect of viral enzymes.

**Figure 2:**
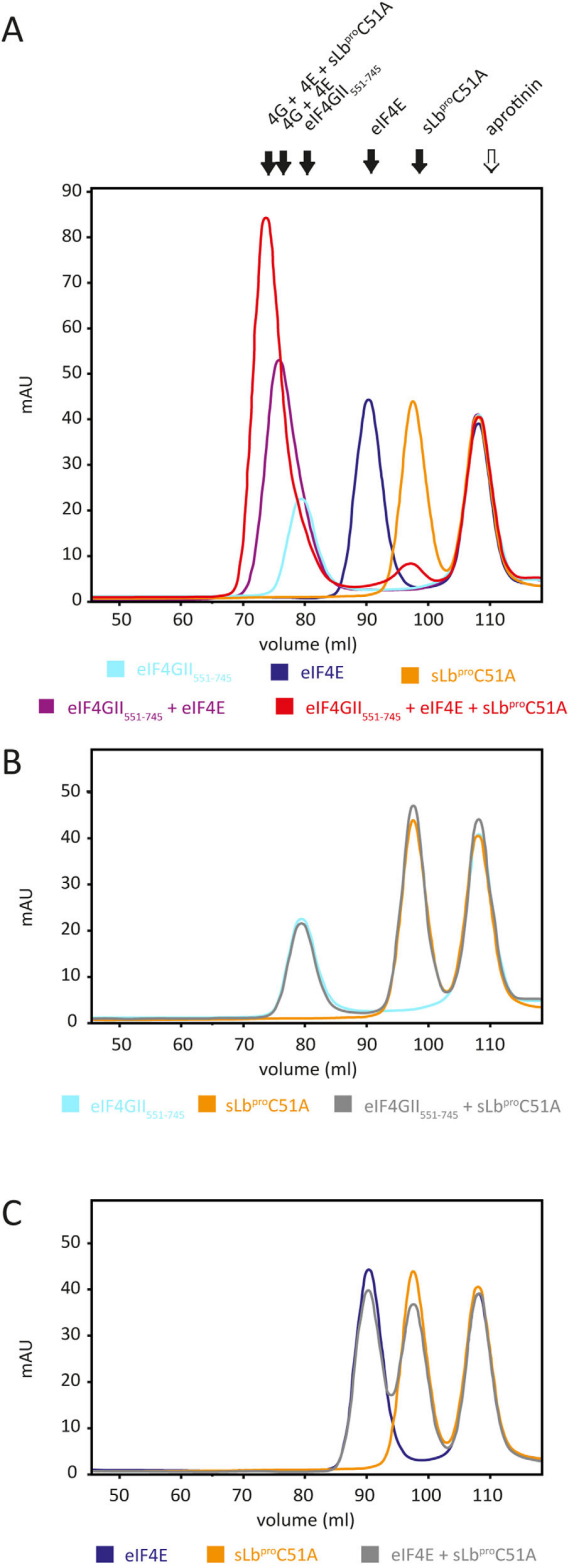
eIF4GII_551–745_ forms a ternary complex with eIF4E and sLb^pro^C51A. Complex formation was analysed via SEC on a HiLoad 16/60 Superdex 200 prep grade column together with aprotinin (6.5 kDa) as an internal standard. 0.5 mg of the individual or complexed proteins were analysed together with 1 mg of aprotinin. Complex formation of two or more proteins was performed by incubation at 4°C for 10 minutes. (A) eIF4GII_551–745_, eIF4E and sLb^pro^C51A (B) eIF4GII_551–745_ and sLb^pro^C51A (C) eIF4E and sLb^pro^C51A. Stable complexes (under conditions of SEC) were observed between eIF4GII_551–745_ and eIF4E as well as between eIF4GII_551–745_, eIF4E and sLb^pro^C51A (both A), but not between eIF4GII_551–745_ and sLb^pro^C51A in the absence of eIF4E (B) and eIF4E and sLb^pro^C51A in the absence of eIF4GII_551–745_ (C).

Why is picornaviral cleavage of eIF4GII_551–745_ in the presence of eIF4E more efficient? To answer this question, we first characterised the complexes formed by sLb^pro^ with eIF4GII_551–745_ and eIF4E. We used the proteolytically inactive variant sLb^pro^C51A (alanine in place of the catalytic cysteine)^28^ and soluble, recombinant murine eIF4E purified from *E.coli* lysates; eIF4E refolded from insoluble material may not always be homogeneous.^22^ eIF4GII_551–745_ and eIF4E form a stable eIF4GII_551–745_/eIF4E complex [Fig. 2(A)] with a dissociation constant (*K*_D_) of 40 n*M*, as determined by ITC (Table 1, Supporting Information Fig. S4). This value is 10 fold higher than that reported for the interaction of human eIF4E with human eIF4GI (residues 557‐646) and yeast eIF4E with eIF4GI (residues 393–490).^22^, ^23^ This may be due to differences in experimental procedures or may reflect variations in the amino acid sequences of the two isoforms. Size‐exclusion chromatography (SEC) analysis showed that sLb^pro^C51A forms a ternary complex with eIF4GII_551–745_/eIF4E [Fig. 2(A)] with a *K*_D_ between 20‐40 n*M* as measured by ITC (Table 1). sLb^pro^C51A, in contrast, does not form a complex with either eIF4GII_551–745_ or eIF4E that is stable enough to be detected by SEC (Fig. 2, B and C) or ITC (data not shown).

**Table 1:** Binding Constants (*K*_D_, µ*M*) for the Interactions of FMDV sLb^pro^ and HRV2 or CVB4 2A^pro^ with eIF4GII_551–745_ and eIF4E as Determined by ITC

Cell	Ligand	Δ*S* ^0^ [cal mol^−1^ deg^−1^]	Δ*G* [kcal mol^−1^]	*K*_D_ (µ*M*)
eIF4E	eIF4GII_551–745_	−18 ± 9.5	−1.1 ± 0.05	0.042 ± 0.004
eIF4GII_551–745_/eIF4E	sLb^pro^C51A	−3.3 ± 3.5	−1.0 ± 0.04	0.033 ± 0.01
eIF4E	HRV2 2A^pro^C106S	−46 ± 1.9	−13 ± 9.2	12 ± 5.1
eIF4GII_551–745_	HRV2 2A^pro^C106S	−7 ± 16	−7.4 ± 4.2	75 ± 12
eIF4GII_551–745_/eIF4E	HRV2 2A^pro^C106S	−12 ± 2.9	−20 ± 8.7	5.1 ± 1.8
eIF4GII_551–745_	CVB4 2A^pro^C110A	−32 ± 18	−15 ± 4.9	19 ± 2.1
eIF4GII_551–745_/eIF4E	CVB4 2A^pro^C110A	−15 ± 9.6	−9.7 ± 6.9	19 ± 12

The average and standard deviation were calculated from three experiments.

The stoichiometry of the eIF4GII_551–745_/eIF4E complex and the eIF4GII_551–745_/eIF4E/sLb^pro^C51A complex were determined by SEC multi‐angle laser light scattering (SEC‐MALLS) to be 1:1 and 1:1:1, respectively (Table 2). Mutations in sLb^pro^ known to impair cleavage on full‐length eIF4GI such as C133S, Q185R/E186K or the triple mutant C133S/Q185R/E186K impaired eIF4GII_551–745_ cleavage; ternary complexes between these mutants and the eIF4GII_551–745_/eIF4E complexes were not formed (Supporting Information Fig. S5).

**Table 2:** Molecular Mass of Indicated Proteins and Protein Complexes Determined by SEC‐MALLS

Protein	Apparent molecular mass (SEC‐MALLS)	Stoichiometry
sLb^pro^C51A	19	1
eIF4GII_551–745_	25	1
eIF4E	30–37	1
eIF4GII_551–745_/eIF4E	58–61	1:1
eIF4GII_551–745_/eIF4E/sLb^pro^C51A	68–70	1:1:1
HRV2 2A^pro^C106S	34	2
eIF4E/HRV2 2A^pro^C106S (overnight)	38–40	1:1
eIF4GII_551–745_/eIF4E/HRV2 2A^pro^C106S	104–106	?
CVB4 2A^pro^C110A	19	1
eIF4GII_551–745_/eIF4E/CVB4 2A^pro^C110A	66–71	1:1:1

### Human eIF4GII_551–745_ remains intrinsically disordered when bound to eIF4E and sLb^pro^

**Figure 3:**
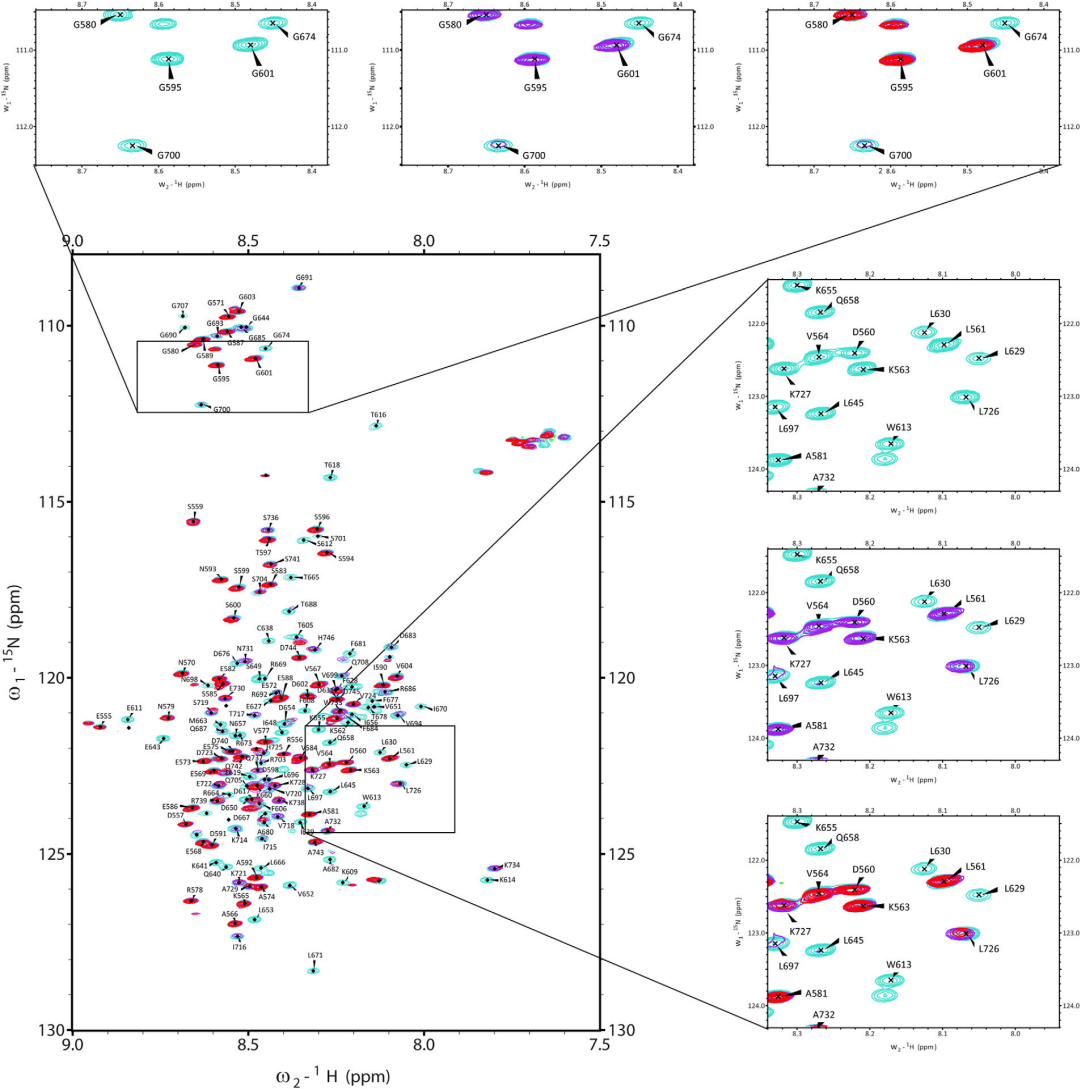
2D ^1^H‐^15^N HSQC spectra of ^15^N labelled eIF4GII_551–745_. Spectra are overlaid for comparison: native eIF4GII_551–745_ (cyan), 1:1 complex of eIF4GII_551–745_ and eIF4E (purple) and 1:1:1 complex eIF4GII_551–745_, eIF4E and sLb^pro^C51A (red). Complexes were purified using SEC and concentrated to 0.7 m*M*. The narrow shift dispersion of cross‐peaks in the ^1^H dimension indicates the lack of a defined tertiary structure for eIF4GII_551–745_. Residues attenuated or disappearing upon the addition of the eIF4E or the sLb^pro^C51A are involved in or affected by complex formation. Signals discussed in the text are shown in insets.

To examine how eIF4GII_551–745_, eIF4E and sLb^pro^ proteins interact, we tried to grow crystals of the eIF4GII_551–745_/eIF4E/sLb^pro^C51A complex for X‐ray diffraction; to date, our efforts have remained unsuccessful, leading us to employ nuclear magnetic resonance (NMR). Accordingly, eIF4GII_551–745_ was isotope labelled with nitrogen (^15^N) or doubly labelled with nitrogen and carbon (^15^N/^13^C) expressed in *E. coli* and purified. High quality NMR spectra of eIF4GII_551–745_, with good signal to noise ratio, were observed at 4°C (Fig. 3) in the assay buffer used to demonstrate stimulation of eIF4GII_551–745_ cleavage by eIF4E [Fig. 1(B–E)]. Spectra were characterised by low ^1^H shift dispersion and resulting severe peak overlap (Fig. 3), indicative of an intrinsically disordered protein (IDP). Nevertheless, sequential backbone signal assignment was possible for 158 (88%) of the 180 non‐proline residues of eIF4GII_551–745_ [Figs. 1(A) and 3].

Analysis of secondary shifts by SSP^30^ and CSI^31^ software confirms that eIF4GII_551–745_ is indeed an IDP largely devoid of regular secondary structure. Furthermore, exploratory CPMG relaxation dispersion experiments of eIF4GII_551–745_ do not reveal any clear dispersion profiles excluding the presence of either a global folded–unfolded equilibrium or substantial contributions from global conformational dynamics on a [μs‐ms] timescale. In addition, certain residues in the eIF4E binding site (residues 620–626) are broadened beyond detection in eIF4GII_551–745_; this may be indicative of [µs‐ms] conformational exchange due to transient local secondary structure formation and/or fluctuating long‐range contacts.

Very few (^1^H,^15^N) chemical shift perturbations were observed between the spectra of eIF4GII_551–745_ and the eIF4GII_551–745_/eIF4E complex, indicating that the binding of eIF4E did not induce any readily observable structural changes in the conformation of eIF4GII_551–745_. Instead, binding of eIF4E to eIF4GII_551–745_ markedly broadens signals of 72 residues (606‐677) and attenuates, albeit to a lesser extent, the signals of residues from 678‐735 (further away from the eIF4E binding site).

**Figure 4:**
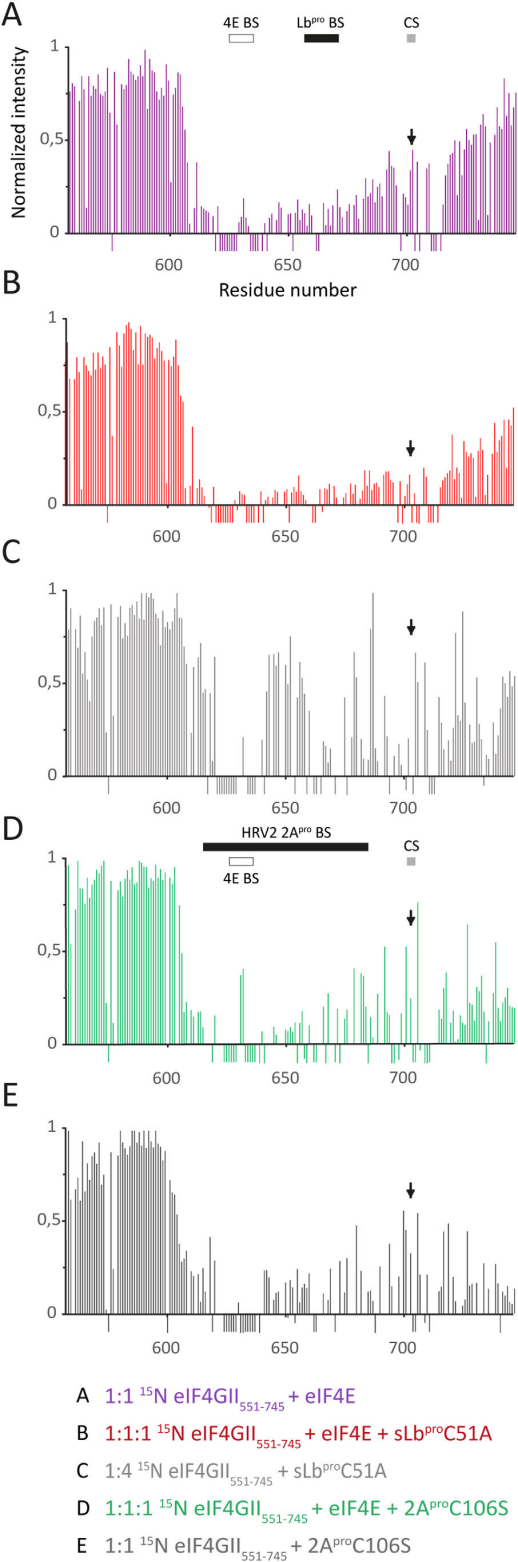
Effect of the addition of eIF4E, sLb^pro^C51A or HRV2 2A^pro^C106S on the signal intensities of the ^15^N HSQC spectrum of eIF4GII_551–745_. A native spectrum of ^15^N eIF4GII_551–745_ was recorded before every titration experiment and its intensities compared to those obtained after addition of the indicated protein(s). Normalized intensities of every assigned residue were calculated by dividing intensities by those of the control spectrum. Residues for which no amide backbone assignment value could be attributed are shown with a value of −0.1. The white boxes represent eIF4E binding site (BS), short black box the Lb^pro^ BS, long black box the HRV2 2A^pro^ BS and grey boxes represent the picornaviral cleavage sites (CS). (A) ^15^N eIF4GII_551–745_ and eIF4E complex purified via SEC. (B) ^15^N eIF4GII_551–745_/eIF4E/sLb^pro^C51A complex purified via SEC. (C) Titration experiment of ^15^N eIF4GII_551–745_ and sLb^pro^C51A. (D) ^15^N eIF4GII_551–745_/eIF4E/HRV2 2A^pro^C106S complex purified via SEC. (E) Titration experiment of ^15^N eIF4GII_551–745_ and HRV2 2A^pro^C106S.

Global changes in the signal intensities in ^15^N heteronuclear single quantum coherence (HSQC) spectroscopy spectra of eIF4GII_551–745_ induced by complex formation are compared in Figures 3 and 4(A,B). The reasons for the signal broadening in the eIF4GII_551–745_/eIF4E are diverse, including conformational exchange and dynamics as well as some increase in the molecule mass of the resulting particle. The dynamic behavior of the intrinsically disordered eIF4GII_551–745_ is complex and still under investigation. Conformational dynamics and exchange between different states of eIF4GII_551–745_ can occur within the free or the bound form or between the two forms as has been observed for other IDPs such as the eIF4E‐binding protein 4E‐BP2 that binds to eIF4E through an extended binding interface.^32^ The lack of observable ^15^N relaxation dispersion effects would suggest that dynamics in free eIF4GII_551–745_ are probably fast on an NMR time scale. In contrast, the low μ*M* affinity of the eIF4GII_551–745_/eIF4E complex implies that conformational exchange between free and eIF4E bound eIF4GII is likely to be slow on the NMR time scale, as indicated by the complete absence of a second set of peaks for the bound form, again as was observed for 4E‐BP2.^32^ In summary, the direct observation of the bound eIF4GII_551–745_ species is precluded by its dynamics and the conformational heterogeneity of a ‘fuzzy’ and extended ensemble, most likely interfacing with a large region on the surface of eIF4E. Nevertheless, we were able in the present study to use signal attenuation as a qualitative indication of the binding interaction.

From the above NMR experiments, we conclude that human eIF4GII_551–745_ remained intrinsically unstructured in complex with murine eIF4E and the resulting complex can be described as ‘fuzzy’ complex. This contrasts with the situation with the yeast fragment eIF4GI_393–490_, for which the NMR data indicates that this region of eIF4GI forms an ordered ring around the N‐terminus of yeast eIF4E.^23^ In the crystal structure of eIF4E with an eIF4GII peptide^14^ corresponding to residues 622‐633, a short α‐helix was observed. In HNCA experiments (data not shown) using eIF4E and eIF4GII_551‐700_, no such α‐helix formation was noted for the observable residues (627‐631), indicating a further difference to the yeast system. Interestingly, in our system, this region which comprises the canonical Y(X)4LΦ binding motif displays some consistent, but small secondary shifts, indicative of transient α‐helix formation. SSP^30^ software reveals a small (ca. 0.18) helical propensity for this region in an otherwise unstructured protein; this is consistent with a nascent α‐helix.

In the eIF4GII_551–745_/eIF4E/sLb^pro^C51A complex, signals between residues 606‐735 were further reduced in intensity; in addition, signals from 735 onwards were attenuated (Fig. 4, compare A and B). This again indicates complex formation, with the binding of sLb^pro^ to the eIF4GII_551–745_/eIF4E complex affecting residues in both its binding and cleavage sites. Again, no evidence for discrete conformational changes through (^1^H, ^15^N) chemical shifts perturbations were observed. The specific changes in the eIF4GII_551–745_/eIF4E/sLb^pro^C51A complex induced by sLb^pro^ binding are illustrated by signals from six glycine backbone amides (Fig. 3, upper panel). The signal intensities of G580, G595 and G601 remain largely unchanged. However, in the eIF4GII_551–745_/eIF4E complex, the signal of G700 is reduced by about 50% and that of G674 is reduced beyond detection. In the eIF4GII_551–745_/eIF4E/sLb^pro^C51A complex, the signal of G700, the residue just prior to the sLb^pro^ scissile bond, is now additionally absent, indicating an interaction with this residue. Together, these selected spectral changes indicate highly specific interactions between eIF4GII_551–745,_ eIF4E and sLb^pro^.

Reductions in signal intensities in the spectrum of eIF4GII_551–745_ were also induced by the addition of the sLb^pro^C51A alone [Fig. 4(C)]. They were, however, much less focussed and less pronounced than those seen with eIF4E, presumably as a consequence of the lower affinity between eIF4GII and sLb^pro^. Regions affected included 615‐630 (the eIF4E binding site), 655‐670 (the sLb^pro^ binding site) and residues 680‐702 (including the sLb^pro^ cleavage site).

### HRV2 2A^pro^ and CVB4 2A^pro^ form a stable complex with eIF4GII_551–745_/eIF4E

**Figure 5:**
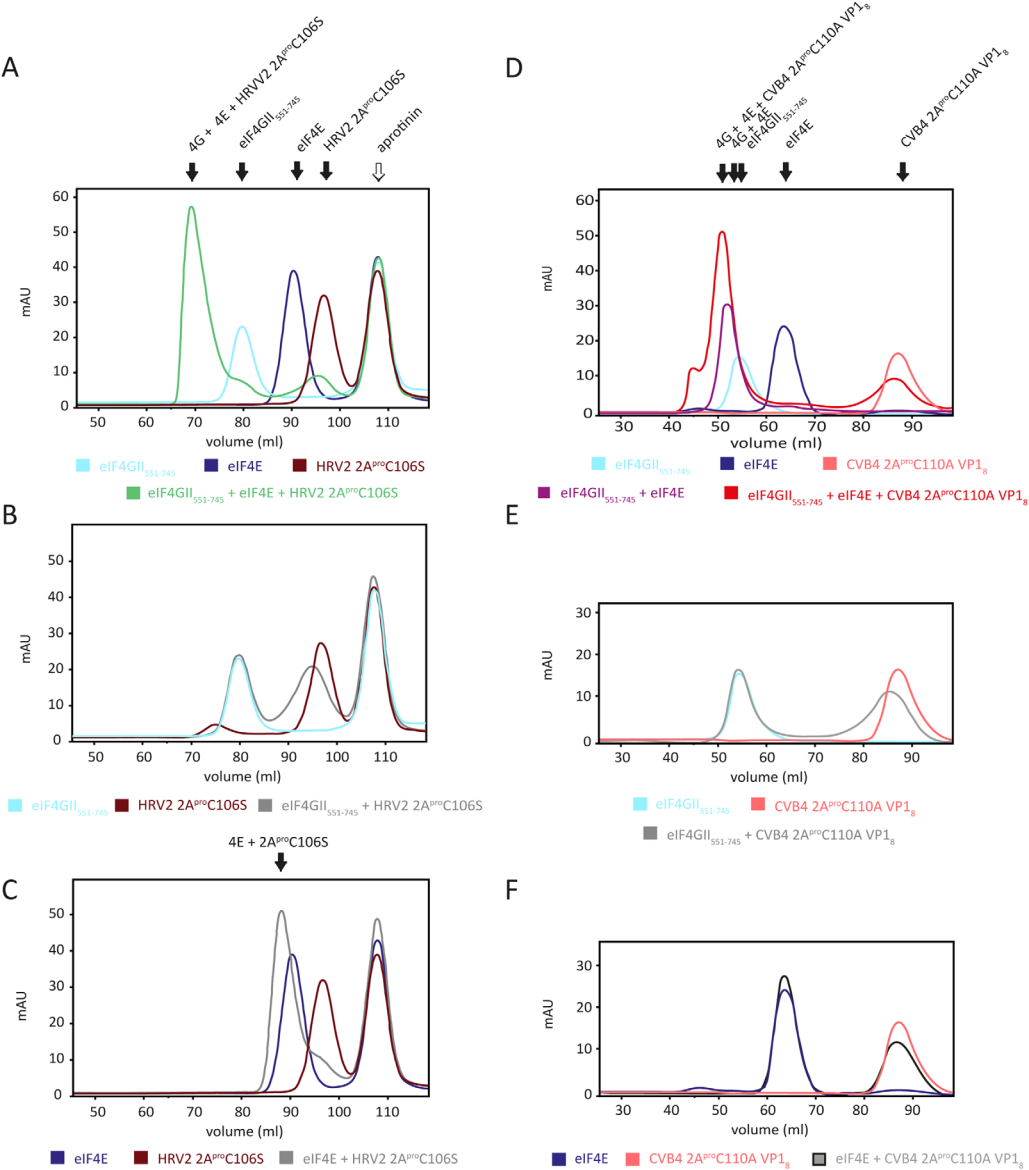
eIF4GII_551–745_ also forms a complex with eIF4E and HRV2 2A^pro^C106S or CVB4 2A^pro^C110A. Complex formation was analysed via SEC on a HiLoad 16/60 Superdex 200 prep grade column (for HRV2 2A^pro^) or on a HiLoad 16/60 Superdex 75 prep grade column (for CV 2A^pro^) together with aprotinin (6.5 kDa) as an internal standard. 0.5 mg of the individual or complexed proteins were analysed together with 1 mg of aprotinin. Complex formation of two or more proteins was performed by incubation at 4°C for 10 minutes. (A) eIF4GII_551–745_, eIF4E and HRV2 2A^pro^C106S (B) eIF4GII_551–745_ and HRV2 2A^pro^C106S (C) eIF4E and HRV2 2A^pro^C106S (D) eIF4GII_551–745_, eIF4E and CVB4 2A^pro^C110A (E) eIF4GII_551–745_ and CVB4 2A^pro^C110A and (F) eIF4E and CVB4 2A^pro^C110A. Stable complexes were observed between eIF4GII_551–745_, eIF4E and HRV2 2A^pro^C106S (A), eIF4E and 2A^pro^C106S (C) and eIF4GII_551–745_, eIF4E and CVB4 2A^pro^C110A (D) but not between eIF4GII_551–745_ and HRV2 2A^pro^C106S in the absence of eIF4E (B), eIF4GII_551–745_ and CVB4 2A^pro^C110A in the absence of eIF4E (E) or eIF4E and CVB4 2A^pro^C110A in the absence of eIF4GII_551–745_ (F).

We now examined whether the proteolytically inactive HRV2 2A^pro^C106S^24^ and CVB4 2A^pro^C110A with a N‐terminal extension of eight VP1 residues^33^ proteins could also form a ternary complex with eIF4GII_551–745_/eIF4E. For HRV2 2A^pro^C106S, an eIF4GII_551–745_/eIF4E/HRV2 2A^pro^C106S complex could be detected by NMR [Fig. 4(D)] and SEC [Fig. 5(A)]; no complex with eIF4GII_551–745_ was observed [Fig. 5(B)]. Examination of the complexes formed by 2A^pro^C106S or sLb^pro^C51A by NMR and SEC revealed appreciable differences. Thus, in the absence of eIF4E, addition of the HRV2 2A^pro^C106S to eIF4GII_551–745_ [Fig. 4(E)] broadens the signals of residues 638‐658 (part of the 2A^pro^ binding site) and the C‐terminal region of eIF4GII_551–745_ (residues 735‐745) to a greater extent than sLb^pro^. This is in line with the larger molecular weight of HRV2 2A^pro^ (it behaves as a dimer of 34 kDa, Table 2)^34^ observed in SEC‐MALLS and its apparently higher affinity to eIF4GII_551–745_; a *K*_D_ of 86 µ*M* was measured by ITC (Table 1). In contrast, eIF4GII_551–745_/eIF4E/2A^pro^C106S complex formation with the 2A^pro^C106S led to a less dramatic loss of signal intensities of eIF4GII than that observed with sLb^pro^C51A.

Two further differences between the complexes observed with 2A^pro^C106S and sLb^pro^C51A were also observed. First, 2A^pro^C106S also formed a stable eIF4E/HRV2 2A^pro^ complex with eIF4E alone (*K*_D_ 8 µ*M* as measured by ITC, Table 1), but not with eIF4GII_551–745_ [Fig. 5(C)]. Second, the affinity for the interaction of eIF4GII_551–745_/eIF4E with 2A^pro^C106S was approximately 100 times lower than that determined for the sLb^pro^C51A (Table 1). These differences are reflected in the slower rates *in vitro* [Fig. 1(B,E)] and *in vivo* of cleavage of eIF4GI observed for HRV2 2A^pro^ than for Lb^pro^.^3^, ^35^, ^36^

The inactive CVB4 2A^pro^C110A could also form a complex with the eIF4GII_551–745_/eIF4E complex [Fig. 5(D)]. However, in contrast to the HRV2 enzyme, the CVB4 2A^pro^ behaves as a monomer (Table 2) and does not form a stable complex with either eIF4E or eIF4GII_551–745_. [Fig. 5(E,F)]. Furthermore, no interaction between eIF4E and CVB4 2A^pro^C110A could be measured by ITC. In contrast, the CVB4 2A^pro^ interacts in ITC with the eIF4GII_551–745_ alone (Table 1).

### sLb^pro^ also interacts directly with eIF4E

**Figure 6:**
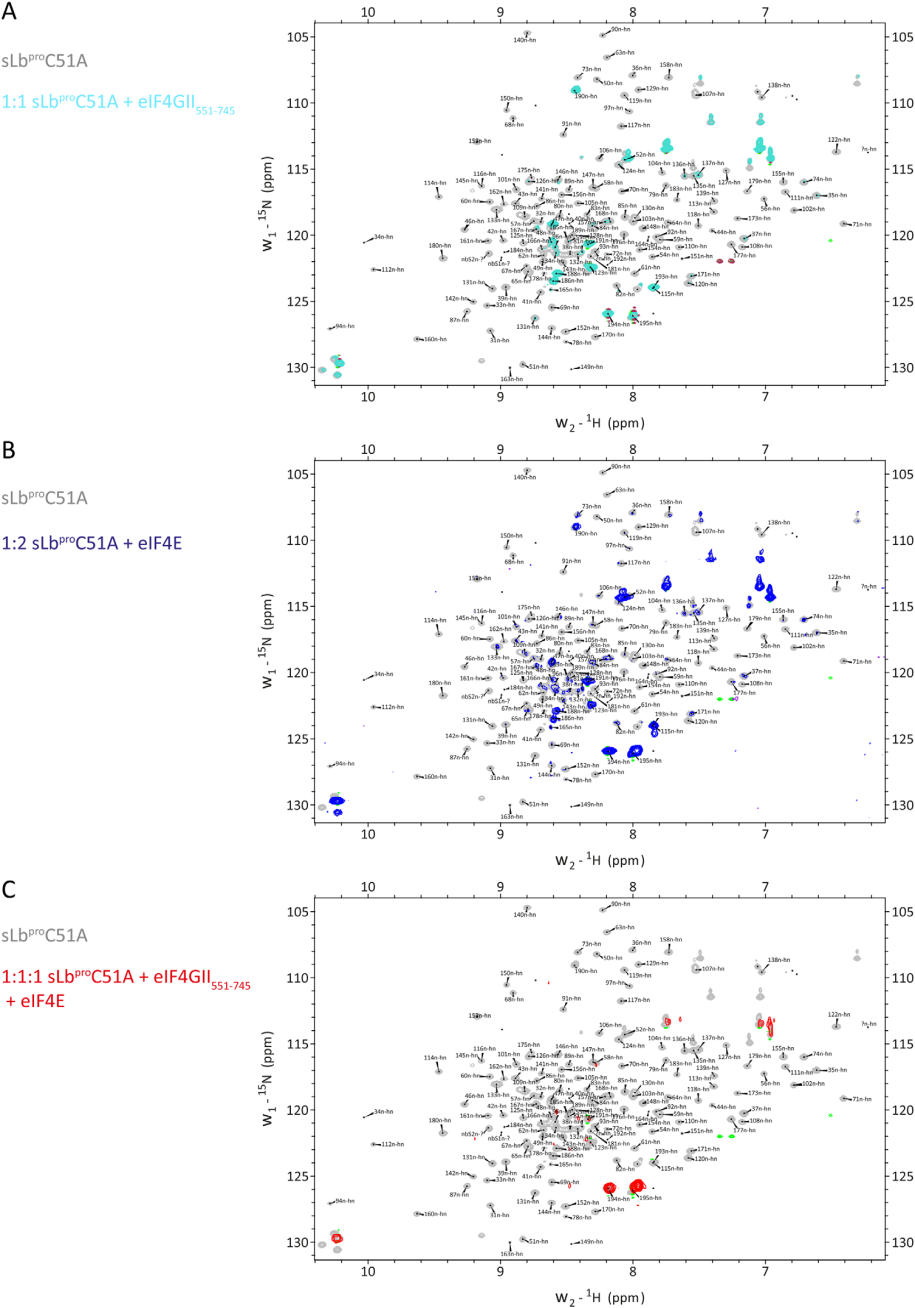
Overlaid spectra from 2D ^15^N HSQC spectroscopy of native sLb^pro^C51A (grey), 1:1 sLb^pro^C51A and eIF4GII_551–745_ (light blue), 1:1 sLb^pro^C51A and eIF4E (dark blue) and 1:1:1 sLb^pro^C51A, eIF4GII_551–745_ and eIF4E (red). The eIF4GII_551–745_/eIF4E/sLb^pro^C51A complex was purified using SEC and concentrated, whereas for binary complex formation the individual proteins were titrated into ^15^N sLb^pro^C51A. The assignments of the ^15^N signals have been assigned previously^27^ (BMRB Entry 15278). As a result of complex formation most residues of sLb^pro^C51A disappear upon the addition of the eIF4GII_551–745_ or the eIF4E, since a globular, folded protein is more strongly affected by binding to an unstructured protein with a comparatively ‘puffed up’ shape. Due the resulting increased hydrodynamic drag of the complex the relaxation rates of the residues involved in the binding event are globally enhanced.

The sLb^pro^ does not form a stable complex with eIF4E; nevertheless, in view of the results with the HRV2 2A^pro^, we decided to search for similar interactions in the complex eIF4GII_551–745_/eIF4E/sLb^pro^. Accordingly, we examined the interaction of uniformly ^15^N labelled sLb^pro^C51A/C133S/Q185R/E186K with eIF4GII_551–745_ alone. This mutant, which fails to form a tight complex with eIF4G/eIF4E and whose eIF4G cleavage reaction is not stimulated by eIF4E [Supporting Information Fig. S5(C,F)], makes the same interactions with eIF4GII_551–745_ as the wild‐type (Supporting Information Fig. S6), indicating that the CTE is not required for interaction with eIF4GII_551–745_. The C‐terminus of sLb^pro^ (residues 183–195) is flexible and protrudes from the globular domain^27^; therefore, we hypothesised that this region may be interacting with eIF4E. To examine this notion, we measured the ^15^N‐HSQC spectra of uniformly ^15^N labelled sLb^pro^C51A in the presence of 1:1 molar ratios of eIF4GII_551–745_, eIF4E and as part of the eIF4GII_551–745_/eIF4E/sLb^pro^C51A complex (Fig. 6). In all experiments, the signals of ^15^N sLb^pro^C51A were dramatically attenuated, indicating the formation of higher molecular weight complexes. This extensive line broadening precluded precise measurement of relaxation rates and quantification of exchange contributions.

With just eIF4GII_551–745_ added, strong signal attenuation was observed across the entire sLb^pro^ globular domain, but not for C‐terminal residues 186‐195. The line broadening effect is global, indiscriminate and diffuse, which resembles the behaviour of proteins with long and unstructured extensions.^37^ With just eIF4E added, the line broadening is more pronounced; thus, intensities across the entire protein, including the C‐terminal residues 183‐191 are attenuated, indicating an interaction with low specificity, although heterogeneity and exchange dynamics may also contribute to this effect. In the eIF4GII_551–745_/eIF4E/sLb^pro^C51A complex, essentially all amide signals of sLb^pro^C51A were lost, except for the very C‐terminal residues 194 and 195. As the effect is widespread across the whole surface of sLb^pro^C51A, it probably originates from the increase in particle size as well as including a contribution from exchange dynamics.

These results provide evidence for weak, low‐to intermediate affinity interactions (too low to be accessible by ITC) between sLb^pro^C51A and eIF4E that include its C‐terminal residues with *K*_D_ values in the mid to high micromolar range (estimated by NMR, as the complex appears to still be in ‘fast exchange’ on the NMR timescale); these interactions help form and stabilise the triangular eIF4GII_551–745_/eIF4E/sLb^pro^C51A complex, that is one with pair‐wise contacts between all three pairs of proteins as defined by Popov.^38^

### C‐terminal residues of sLb^pro^ interact with eIF4E

Overall, the above NMR data indicate differing structural contexts and environments for eIF4GII_551–745_ residues 551‐605 and 606‐745 as well as the close proximity and direct contacts of the C‐terminal sLb^pro^ residues to the eIF4E in the eIF4GII_551–745_/eIF4E/sLb^pro^C51A complex. To investigate these insights further, we used paramagnetic relaxation enhancement (PRE) to sensitively probe local compaction by intramolecular PREs and transient tertiary contacts by intermolecular PREs. A paramagnetic spin‐label (*S*‐(1‐oxyl‐2,2,5,5‐tetramethyl‐2,5‐dihydro‐1H‐pyrrol‐3‐yl)methyl methanesulfonothioate, MTSL), reaching out up to approximately 20 Å, was first attached to residue C638 of eIF4GII_551–745_ and the NMR spectra of this protein alone were recorded. Most signals from residues 606‐715 (which is also the region of interaction with eIF4E and sLb^pro^) were lost, with those from 715‐745 being affected to a lesser degree [Supporting Information Fig. S7(A)]. Thus, the residues whose NMR relaxation properties are affected by eIF4E or sLb^pro^ are indeed located in a fairly compact globular core of approximately 20 Å which is still partially unstructured and located in an otherwise even more unstructured protein. The spin‐label was then attached to residue C133 of sLb^pro^C51A/C125S/C153S and the NMR spectra of eIF4GII_551–745_ were measured in the presence of the spin‐labelled sLb^pro^C51A/C125S/C153S [Fig. S7(B)]. A strong attenuation of signals in the eIF4E binding site (628–631) of eIF4GII_551–745_ and a partial signal attenuation of residues (638‐655) between the eIF4E binding site and the Lb^pro^ binding site of 50% was observed [Fig. S7(B)], again indicating that these residues are within 20 Å of residue C133 of sLb^pro^.

**Figure 7:**
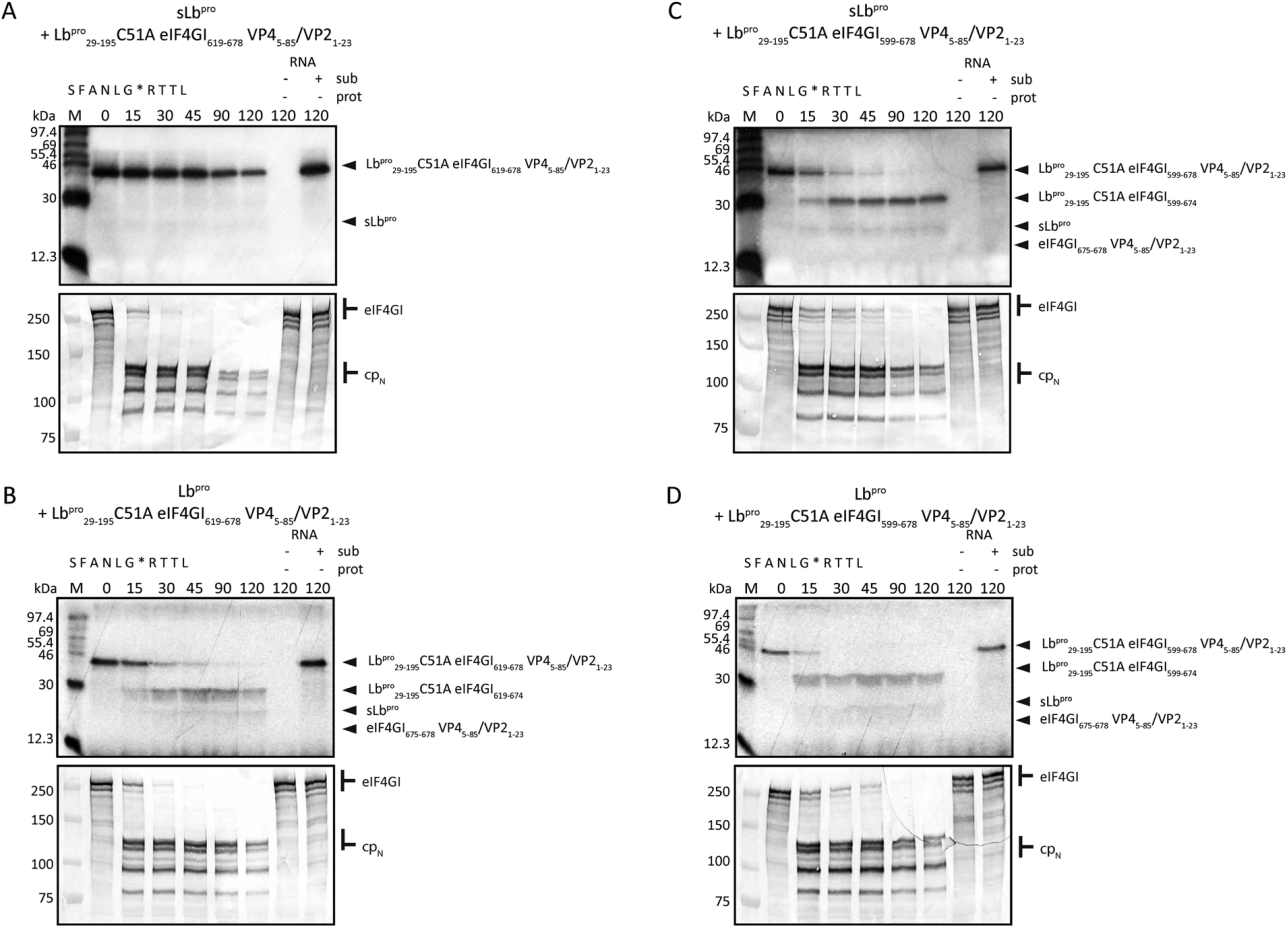
*In vitro* translation assays of the intermolecular processing of Lb^pro^C51A eIF4GI_619‐678_ VP4/VP2 (A and C) or Lb^pro^C51A eIF4GI_599‐678_ VP4/VP2 (B and D) by sLb^pro^ (A and B) and Lb^pro^ (C and D). Rabbit reticulocyte lysate (RRL) was programmed with 14 ng/µL RNA coding for Lb^pro^C51A eIF4GI_599‐678_ VP4_5‐85_/VP2_1‐23_ (eIF4GI_599‐678_ sequence with the eIF4E and the Lb^pro^ binding site) or Lb^pro^C51A eIF4GI_619‐678_ VP4_5‐85_/VP2_1‐23_ (eIF4GI_619‐678_ sequence lacking the eIF4E binding site) comprising the eIF4GI cleavage site SFANLG*RTTL. Precursor substrate protein was translated for 20 minutes in the presence of [^35^S]‐Met before adding 14 ng/µL active sLb^pro^ or Lb^pro^ RNA in the presence of unlabelled Met to the reaction. 10 µL aliquots were taken at the indicated time points and cleavage of the substrate protein was analysed on 17.5% SDS gels followed by autoradiography (upper pictures). Uncleaved precursors Lb^pro^C51A eIF4GI_599‐678_ VP4/VP2 or Lb^pro^C51A eIF4GI_619‐678_ VP4/VP2 as well as cleavage products Lb^pro^C51A eIF4GI_599‐674_, Lb^pro^C51A eIF4GI_675‐678_ VP4_5‐85_/VP2_1‐23_ or Lb^pro^C51A eIF4GI_619‐674_, Lb^pro^C51A eIF4GI_675‐678_ VP4_5‐85_/VP2_1‐23_ are indicated. To observe cleavage on endogenous eIF4GI in the RRLs, 10 µL aliquots were analysed on 6% SDS gels followed by Western blotting using serum to detect the N‐terminal part of the eIF4GI protein (lower pictures). Uncleaved eIF4GI and cleavage products (CP_N_) are indicated. Negative controls without any RNA (−sub, −prot) or comprising only RNA of the substrate protein (+sub, −prot) are shown at the right of each gel. Protein standards are shown on the left.

We also wished to investigate whether the presence of the eIF4E binding site would be required for cleavage by sLb^pro^ at the wild‐type eIF4G cleavage site. For this, we replaced 10 residues of the cleavage site between Lb^pro^ and VP4 with those of the eIF4GI cleavage site.^16^ This site is cleaved by full‐length Lb^pro^ but not by sLb^pro^ when the encoded protein is synthesised in RRLs. However, when residues 599–678 of eIF4GI N‐terminal of the cleavage site are included in the substrate, sLb^pro^ was also able to cleave this substrate.^16^ To examine whether sLb^pro^ could cleave when the eIF4E binding site was removed, we constructed a DNA fragment containing residues 29–195 of sLb^pro^C51A, residues 618–678 of eIF4GI (*i.e*., lacking the eIF4E binding site but maintaining the sLb^pro^ binding site), 5–85 of VP4 and 1‐23 of VP2. RNA from this construct was translated in RRLs in the presence of ^35^S‐methionine for 20 minutes followed by addition of unlabelled methionine and RNA for sLb^pro^. No cleavage of the synthetic polyprotein/eIF4GI substrate by sLb^pro^ was observed over a period of 120 minutes [Fig. 7(A), upper panel]. The synthesis of active sLb^pro^ in RRLs was demonstrated by the cleavage of endogenous eIF4GI detected by immunoblotting of the samples [Fig. 7(A), lower panel]. As a further control for sLb^pro^ activity, we tested the 599–678 substrate containing the eIF4E binding site reported in Steinberger *et al*.^16^ and showed that it could be cleaved by sLb^pro^ [Fig. 7(B), upper panel]. To control that the eIF4GI construct bearing residues 619–678 of the eIF4GI sequence could serve as a substrate, we incubated it with Lb^pro^ and showed that it could be cleaved to the same extent as the 599–678 substrate [Fig. 7(C,D), upper panels]. In all experiments, the synthesis of active Lb^pro^ and sLb^pro^ was confirmed by endogenous eIF4GI cleavage (Fig. 7, lower panels).

## Discussion

The ability of enteroviruses and aphthoviruses to replicate successfully depends on the ability of the virus to shut down the synthesis of host proteins. FMDV lacking Lb^pro^ fail to spread in the infected animal^8^ whereas enteroviruses with mutations in 2A^pro^ have small plaque phenotypes in cell culture.^39^, ^40^ It has long been recognised that, although the host cell shut‐off achieved through the cleavage by Lb^pro^ and 2A^pro^ is the same, the variations in proteinase structure, locations of the cleavage sites and the times of onset of cleavage^3^, ^41^, ^42^, ^43^, ^44^ imply that the interactions with the eIF4GII/eIF4E complex differ considerably.

Several observations documented that the eIF4GII_551–745_ fragment analysed here behaved as the full‐length eIF4G isoforms. First, complex formation between eIF4GII and eIF4E was measured by four techniques (SEC, SEC‐MALLS, ITC (*K*_D_ 40 n*M*) and NMR); mutation of the binding site for eIF4E on eIF4GII_551–745_ eliminated complex formation. The observed *K*_D_ of 40 n*M* compares well with those (around 4 n*M*) obtained for similar, but shorter, fragments of yeast and human eIF4GI.^22^, ^23^ These differences may reflect amino acid differences between the two isoforms. Second, conditions were found in which cleavage of eIF4GII_551–745_ by sLb^pro^ were observed only in the presence of eIF4E, as previously observed.^13^ Third, a stimulation of eIF4GII_551–745_ by eIF4E was observed by both sLb^pro^ and HRV2 2A^pro^; the results shown here extend this observation to the CVB4 2A^pro^.

The major hurdle to the study of the nature of the complexes formed by the proteinases with the initiation factors was the assignment of the NMR signals of the human eIF4GII_551–745_. This was achieved despite the intrinsically unstructed nature of the protein. In contrast to the yeast eIF4GI_393–490_,^23^ human 4GII_551–745_ remains intrinsically unstructured when bound to eIF4E. Indeed, neither discrete conformational changes nor a defined tertiary structure were observed on binding of murine eIF4E to the human eIF4GII_551–745_. Nevertheless, the NMR data for all complexes measured are consistent with the notion that eIF4G and eIF4E form a tight complex in which eIF4E contacts between eIF4GII residues 606 and 678. Whatever the nature of the interactions, this system should prove useful in screening for compounds that interrupt this interaction, a central control point in mammalian protein synthesis.

Given the lack of defined structure observed in eIF4GII_551–745_ in the presence or absence of eIF4E, it is of interest to note that a similar phenomenon was previously observed in the binding of both human 4E‐BP1 and 4E‐BP2 to eIF4E.^32^, ^45^ Both 4E‐BP1 and 4E‐BP2 are IDPs that remain unstructured when bound to eIF4E.^32^, ^45^, ^46^ The binding of the 4E‐BP to eIF4E represents an important control point as it prevents eIF4G isoforms from binding to eIF4E or even displaces bound eIF4G from eIF4E.^47^ Studies of the interaction between eIF4E and 4E‐BP have revealed that both human 4E‐BP2 and 4E‐BP from *D. melanogaster* bind to two sites (*i.e*., in a bipartite manner) on eIF4E^32^, ^47^; for the *D. melanogaster* 4E‐BP, one site is that at which the eIF4G isoforms bind whereas the other is on the lateral face comprising the C‐terminus of helix α1 and loop 2.^47^ It will be of interest to see whether the picornaviral proteinases also use the lateral face for their eIF4E interactions or a different part of the protein.

**Figure 8:**
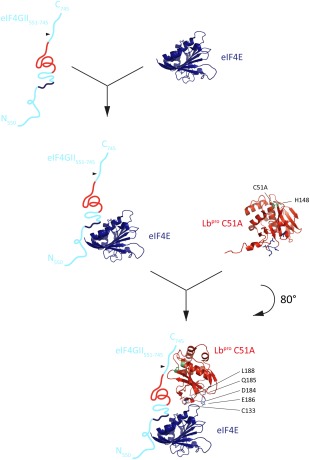
Model of the heterotrimeric triangular interactions of eIF4GII_551–745_, eIF4E and sLb^pro^C51A. Binding sites for eIF4E and sLb^pro^ on the eIF4GII_551–745_ are indicated in purple and red, respectively; a triangle indicates the Lb^pro^ cleavage site. Drawings were created in PyMol.^55^ PDB identifiers: 1QOL, Lb^pro^C51A; 1EJH, eIF4E.

Together, the above experiments indicate that all three proteins in the ternary complexes of eIF4GII_551–745_ with either sLb^pro^C51A, HRV2 2A^pro^C106S or CVB4 2A^pro^C110A interact with each other, forming heterotrimeric complexes that are also termed triangular complexes.^38^, ^48^ The triangular protein‐protein interactions involved in sLb^pro^ host cell shut‐off are summarised in a model (Fig. 8). Residues 606–705 of eIF4GII_551–745_ are shown as a relatively compact, preformed domain to which eIF4E binds. In the absence of structural evidence on the parts of murine eIF4E that interact with eIF4GII, we have drawn the model with eIF4GII contacting the N‐terminus of eIF4E as in the yeast system.^23^ Triangular ternary complex formation is stabilised by the interaction of the globular sLb^pro^ domain with its binding site on eIF4GII and by interactions of residues C133, Q185 and E186 of the C‐terminus (residues marked with side‐chains) with eIF4E. Further support for the importance of this reaction was provided by the experiment in which cleavage by sLb^pro^ at the eIF4GI cleavage site could be made dependent on the presence of the eIF4E binding site on eIF4GI (Fig. 7). Furthermore, the model explains why the presence of eIF4E can drive the processivity of eIF4G isoform cleavage as documented for Lb^pro^ by Ohlmann *et al*.^13^ in RRLs. Further evidence for the importance of eIF4E for the processivity is that, in actively translating ribosomes in RRLs, eIF4GI is efficiently cleaved during the synthesis of both L^pro^ (50% cleavage in 4 min.) and HRV2 2A^pro^ (50% cleavage in 15 min.).^49^ Rau *et al*.^50^ showed that 80% of eIF4GI is present in on the ribosome under such conditions and bound to eIF4E. This suggests that that cleavage of the eIF4G isoforms begins on the ribosome during synthesis of proteins from the RNA of the genome of the infecting particle, thus explaining the efficient cleavage of the isoforms *in vivo*. Indeed, cleavage of the eIF4G isoforms has been observed during PV under conditions that prevented the replication of viral RNA.^4^

The HRV2 2A^pro^C106S also formed a triangular heterotrimeric complex with eIF4GII and eIF4E. However, the affinity of this 2A^pro^ to the eIF4E/eIF4GII complex was lower than that of sLb^pro^. Furthermore, the stoichiometry of the HRV2 2A^pro^C106S complex remained unclear as SLS measurements indicated an apparent molecular mass of 104‐109 kDa (Table 2). This corresponds neither to a 1:1:1 complex nor to any simple multiples of the components. Given this uncertain stoichiometry and the ill‐defined nature of the binding site for HRV2 2A^pro^,^11^ we cannot yet provide a model for HRV2 2A^pro^ analogous to that for sLb^pro^ shown in Figure 8.

A further interesting observation was the formation of a stable complex between HRV2 2A^pro^C106S and eIF4E, as evidenced by SEC, SLS and ITC measurements. This interaction helps to explain why previous attempts to exactly define the binding site of HRV2 2A^pro^ on eIF4GI (600‐674) or eIF4GII [613‐685, Fig. 1(A)] met with little success.^11^ In these experiments, performed in the presence of eIF4E, binding of HRV2 2A^pro^ to eIF4G was lost upon deletion of the eIF4E binding site. Thus, it would appear that eIF4E is acting as a bridge to anchor the HRV2 2A^pro^ to the eIF4G isoforms, indicating a fundamental difference to the model for sLb^pro^ shown in Figure 8.

The apparent molecular mass determined by SLS on eIF4E/2A^pro^C106S complexes formed overnight was 38–40 kDa (Table 2), indicating a 1:1 complex between 2A^pro^C106S and eIF4E. This would require dissociation of the 2A^pro^ dimer observed in solution^34^ (Fig. 5). Evidence for unusual molecular rearrangements in the formation of the eIF4E/2A^pro^C106S complex came when the SLS measurements were made after 10 min of complex formation, with an apparent molecular mass of over 300 kDa being observed (data not shown), compared to the 38–40 kDa observed on overnight complex formation. The dimerization interface for HRV2 2A^pro^ has been predicted to involve amongst others residue C138, involved in a disulphide bridge between monomers in the crystal structure of HRV2 2A^pro^.^15^ Investigation of a mutant HRV2A^pro^ bearing the mutations C106S and C138S showed that the 1:1 complex with eIF4E was formed in this case after 10 min, suggesting that a reduction in the stability of the HRV2 2A dimer favours complex formation with eIF4E. Together, these results suggest that the monomer–dimer equilibrium of HRV2 2A^pro^C106S, together with known propensity of eIF4E to aggregate,^18^ complicates the determination of the stoichiometry of the complex. Whatever the mechanism of complex formation, it will be of interest to investigate the interaction of the proteinases with eIF4E in transfected cells, using for instance fluorescently tagged proteins as has been done to investigate the dimerization state of a vaccinia virus protein.^51^

The CVB4 2A^pro^C110A complex with eIF4GII_551–745_/eIF4E clearly differed from that of HRV2 2A^pro^C106S. The stoichiometry was 1:1:1, as was that of the sLb^pro^. The CVB4 2A^pro^C110A is monomeric, as previously observed by Liebig *et al*.;^34^ the protein did not form a complex with eIF4E alone. In addition, it was the only protein to form a measurable complex with eIF4GII_551–745_ (Table 1). CVB4 2A^pro^ shares the 1:1:1 stoichiometry of binding with sLb^pro^. However, as we have no information on the precise interactions of CVB4 2A^pro^ with eIF4GII_551–745_ and eIF4E, the validity of the model in Figure 8 for CVB4 2A^pro^ remains to be determined. HRV2 and CVB4 2A^pro^ examined have the same fold^15^, ^33^; nevertheless, they share an amino acid identity of about only 40% (Supporting Information Fig. S8), with the majority of the identity in the C‐terminal domain. One might therefore predict that the CVB4 and HRV2 2A^pro^ behave differently toward eIF4E and the eIF4GII/eIF4E complex itself. These mechanistic differences may also explain why HRV2 cleaves both isoforms at the same rate in infected HeLa cells whereas in HRV14 and PV (both of which are more closely related to CVB4 than HRV2) infected HeLa cells, the eIF4GI isoforms are cleaved more rapidly than the eIF4GII isoforms.^35^, ^41^, ^42^ Investigation of the importance of eIF4E interactions on the cleavage of full‐length eIF4G isoforms during infection awaits the development of cell lines over‐expressing recombinant eIF4G that can be infected by picornaviruses.

In summary, the data presented here show that FMDV sLb^pro^, HRV2 2A^pro^ and CVB4 2A^pro^ have each evolved different interactions to interact with the eIF4GII_551–745_/eIF4E complex, even though the viruses are all from the same family and that two of the proteinases share the same reduced chymotrypsin‐fold.

## Materials and Methods

### Materials

^13^C_6_‐D‐glucose and ^15^N‐ammonium chloride were purchased from Sigma‐Aldrich, Germany and Euriso‐Top, France, respectively. For over‐expression of recombinant proteins in *E. coli*, the following vector systems were used: pET11d (Novagen®) was used for all eIF4GII constructs, active sLb^pro^ and sLb^pro^C51A, pET3d (Novagen®, formally known as pET8c) for HRV2 2A^pro^, HRV2 2A^pro^C106S, and CVB4 2A^pro^C110A VP1_8_ and _p_proExHTA (Invitrogen®) for eIF4E.

### Protein expression and purification

Fragments of eIF4GII (all having a C‐terminal HisTag) were expressed in BL21(DE3) grown in LB medium for biochemical analysis or minimal medium (M9) for NMR studies containing ^13^C_6_‐D‐glucose as sole carbon source and ^15^N‐ammonium chloride as sole nitrogen source. Briefly, transformed bacteria were grown in 100 mL M9 medium overnight. 2 L expression cultures were then inoculated with the overnight culture and protein expression was induced at an OD_600_ between 0.5 and 0.6 for 5–8 h at 37°C. Following sonication, the sample was loaded on a 5 mL HisTrap HP (GE Healthcare), equilibrated with 50 m*M* Tris/HCl, 50 m*M* NaCl and 20 m*M* imidazole, washed with five column volumes and eluted with increasing imidazole concentration (500 m*M*). Pooled fractions were dialyzed into assay buffer (20 m*M* Hepes/KOH pH 7.4, 150 m*M* KCl, 1 m*M* EDTA and 5 m*M* DTT). For preparative SEC, a HiLoad 26/60 Superdex 75 pg column was used (GE Healthcare), equilibrated with assay buffer. The samples were concentrated to about 0.5–1 m*M* using Amicon Ultra Centrifugal Devices with a 10 kDa cut‐off (Millipore). For expression of unlabelled eIF4GII, expression was in LB medium and was terminated after 5 h at 37°C.

Expression of sLb^pro^C51A (FMDV residues 29–195) was described previously.^28^ PCR mutagenesis was used to restore the active cysteine residue C51. The expression and purification protocol of active sLb^pro^ and mutants thereof was as described,^27^ with the exception that plasmids were transformed in BL21(DE3)plysE and that assay buffer was used in the last step of purification.

Expression and purification of active HRV2 2A^pro^, HRV2 2A^pro^C106S as well as active CVB4 2A^pro^ and CVB4 2A^pro^C110A VP1_8_ were performed as described previously^33^, ^34^ with the exception that assay buffer was used in the final SEC purification step .

Recombinant murine eIF4E (N‐terminal HisTag) was purified from strain T7 Express (New England Biolabs). Briefly, cells from 4 L were resuspended in 40 mL 50 m*M* Tris/HCl pH 8, 50 m*M* NaCl, 1 m*M* EDTA and 5% glycerol, lysed by sonication and centrifuged at 18,000 rpm for 30 min at 4°C. The supernatant was then loaded onto a 5 mL HisTrap, equilibrated with 50 m*M* Tris/HCl, 50m M NaCl and 20 m*M* imidazole, washed with five column volumes and eluted with increasing imidazole concentration (500 m*M*). Pooled fractions were loaded onto a MonoQ HR 10/100 (GE Healthcare) column, equilibrated with buffer containing 50 m*M* Tris/HCl pH 8.0, 50 m*M* NaCl, 1 m*M* EDTA, 5 m*M* DTT and 5% glycerol, washed with five column volumes and eluted with rising NaCl concentrations (1*M*). Pooled fractions were dialyzed into assay buffer, followed by SEC using a HiLoad® 26/60 Superdex® 75 pg (GE Healthcare).

### In vitro cleavage assays

Cleavage assays were performed using purified recombinant eIF4GII_551–745_, murine eIF4E and FMDV sLb^pro^ wt or mutants. 1 µg of substrate protein (equimolar eIF4GII_551–745_ and eIF4GII_551–745_ or eIF4E alone) were incubated with 1 ng of wt sLb^pro^ or mutant sLb^pro^ in assay buffer at 37°C. After 0, 10, 20, 30, 60, and 90 minutes, reactions were stopped by the addition of SDS‐PAGE 5× loading buffer and heated at 95°C for 5 minutes. Reactions were visualized on a 17.5% SDS‐PAGE gel stained with Coomassie brilliant blue. Gels were captured by a scanner (Canon) and uniform adjustments were performed using Adobe Photoshop. Densitometric analysis was performed by the ImageJ software.

### NMR spectroscopy

Two and three‐dimensional ^1^H ^15^N (^13^C)‐NMR experiments were performed at 4°C on Varian Inova 500 MHz and 800 MHz and Varian Direct Drive 600 MHz spectrometers equipped with 5‐mm triple resonance probes and pulsed field gradients. Samples containing 0.6–1 m*M* ^15^N/(^13^C) eIF4GII_551–745_ or other fragments of eIF4GII in assay buffer and 5‐10% (v/v) D_2_O for field/frequency lock were used for the NMR measurements. For triple resonance experiments 0.1‐0.2% (w/v) NaN_3_ was added to the samples to inhibit bacterial growth. When possible, NMR measurements were performed on binary and ternary complexes that had been purified by SEC. The spectra were processed with NMRPipe^52^ and analysed with Sparky (Goddard, unpublished).

Backbone amide ^1^H^N^, ^15^N, ^13^Cα, ^13^C', and side chain ^13^C^β^ resonances of eIF4GII_551–745_ were obtained by measuring a set of standard three‐dimensional triple resonance experiments:^53^ HNCA, HN(CO)CA, HNCACB, HN(CO)CACB, HN(CA)CO, HNCO. Furthermore, HNN and HNCN experiments were conducted to establish sequential connectivities. As a further aid, resonance assignments of sub‐fragments of eIF4GII (eIF4GII _653‐745_, eIF4GII_551‐700_, eIF4GII_714‐745_) were performed.

The nitroxide spin‐label MTSL was introduced by adding two to three times excess of MTSL to the protein, which was prior separated from DTT by a PD‐10 desalting column (GE Healthcare). After incubation for 2–3 h, the protein was concentrated to 500 µL. Intramolecular paramagnetic relaxation enhancements (PREs) by nitroxide spin‐label MTSL in single‐cysteine C638.MTSL ^15^N eIF4GII_551–745_ or intermolecular PREs caused by unlabelled C133.MTSL sLb^pro^C51A/C125S/C153S were measured as intensity ratios of cross‐peaks in 2D ^15^N HSQC NMR spectra between oxidized and reduced forms. DTT was used to reduce the paramagnetic spin label. Individual cross‐peak intensities were normalized using the most intense peaks in each data set.

### Limited proteolysis

20 µg of eIF4GII_551–745_ was subjected in the presence or absence of 20 µg eIF4E to limited proteolysis in assay buffer at 37°C by the addition of α‐chymotrypsin, trypsin, elastase, papain, subtilisin or endoproteinase Glu‐C to final concentrations of 50, 5, and 0.5 µg/mL. The reaction was stopped after 60 minutes by adding 5× SDS sample buffer and immediately heated at 95°C for 5 min. The samples were analysed on 17.5% polyacrylamide SDS‐PAGE gels.

### Analytical size‐exclusion chromatography

Size‐exclusion chromatography was performed with a HiLoad® 16/60 Superdex® 200 pg (GE Healthcare) column, equilibrated with a assay buffer. 0.5 mg of pure protein or complexed proteins (10 minutes at 4°C under gentle mixing) were injected together with 1 mg of aprotinin (6.5 kDa) using a 2 mL loop and eluted with 1 mL/min flow rate. Protein elution was monitored by following the absorption at 280 nm (mAU).

### Static light scattering

For assessing the molecular weight and oligomeric state of eIF4GII_551–745_, eIF4E, sLb^pro^C51A and 2A^pro^C106S, a Superdex 200 10/300 column was equilibrated with assay buffer. The miniDAWN Trista light scattering instrument (Wyatt Technology, Santa Barbara, CA) was connected and 100 µg sample was injected on the gel‐filtration column. Data analysis was performed using the manufacturer's software ASTRA.

### Studying protein–protein interactions using isothermal titration calorimetry (ITC)

ITC experiments were performed on a MicroCal ITC microcalorimeter. Proteins were dialyzed before the measurement in a buffer containing 20 m*M* Hepes/KOH pH 7.4, 150 m*M* KCl and 1 m*M* EDTA filtered through a 0.22 µm filter. Following thermal equilibration at 25°C, an initial delay of 60 seconds and a single 0.5 µL titrant injection, 20 serial injections of 2 µL of the titrant was added at an interval of 180 seconds into the stirred sample cell (200 µL) at a stirring rate of 750 rpm at 25°C. The protein concentration in the cell was about 10× lower than protein titrant. Data analysis was performed using the Origin software package MicroCal.

### In vitro transcription and translation

*In vitro* transcription reactions were performed as described.^16^, ^54^ *In vitro* translation reactions were performed as described in^16^, ^27^ with an RNA concentration of 14ng/µL.

## Supporting information

Supporting Information

Supporting Information Figure S1

Supporting Information Figure S2

Supporting Information Figure S3

Supporting Information Figure S4

Supporting Information Figure S5

Supporting Information Figure S6

Supporting Information Figure S7

Supporting Information Figure S8
