# Supplementary material for: NMR analysis of the interaction of picornaviral proteinases Lb and 2A with their substrate eukaryotic initiation factor 4GII
Source: Protein Sci. 2015 Oct 4;24(12):1979–96. doi: 10.1002/pro.2807 (PMC4815241; doi:10.1002/pro.2807)
Supplement: Supplementary file 1 — Supporting Information [file PRO-24-1979-s001.docx]

**SUPPLEMENTARY INFORMATION**

**FIGURE S1.**  Amino acid sequence alignment of yeast eIF4GI (p150, Genbank Identifier AAA02757.1) with the human homologues for eIF4G (I and II, Genbank Identifiers Q04637.4 and O43432.2) using the ClustalW2 online tool.^1^ The eIF4GII_551-745_ fragment used in this manuscript is highlighted in dark blue, the *S.cerevisiae* fragment used (393-490) by Gross *et al.* is highlighted in red^2^ and the conserved eIF4E binding site is highlighted in grey. The cleavage sites on human eIF4GI and eIF4GII by the Lb^pro^ or the HRV2 2A^pro^ are depicted with triangles: Lb^pro^ cleavage site on eIF4GI in green and on eIF4GII in black, HRV2 2A^pro^ cleavage site on eIF4GI in yellow and on eIF4GII in red.^3^ The identity of full-length human eIF4GII to human eIF4GI and yeast eIF4GI is 54% and 26%, respectively. The identity of human eIF4GII_551-745_ to the corresponding regions of human eIF4GI and yeast eIF4GI is 48% and 20%, respectively.

**FIGURE S2.** A) *In vitro* cleavage reaction of 5 µM of eIF4GII_551-745_ in the absence or the presence of eIF4E by different concentrations of sLb^pro^. Cleavage reactions were performed at 37°C for the times indicated and samples and proteins visualized with Coomassie blue. B) *In vitro* cleavage of 10 µM eIF4GII_653-745_ by 70 nM of sLb^pro^ in the absence or the presence of 5 µM eIF4E.

**FIGURE S3.** Limited proteolysis of eIFGII_551-745_ in the presence and absence of eIF4E. 20 µg aliquots of eIF4GII_551-745_ in the absence **(A** and **B)** or presence of 20 µg of eIF4E **(C** and **D)** were digested with indicated amounts of the indicated proteases and analysed by SDS-PAGE, followed by staining with Coomassie blue. In addition, eIF4E alone was also subjected to limited proteolysis digestion **(E** and **F)**. eIF4GII_551-745_ was completely cleaved with 5 µg/ml trypsin, elastase and subtilisin in the absence of eIF4E (red circles); however, in the presence of eIF4E, eIF4GII_551-745_ appears to be resistant to cleavage by 5 µg/ml trypsin, elastase and subtilisin, which strongly indicates protection by complex formation.

**FIGURE S4.** Raw data from isothermal titration calorimetry. The top graphs show raw data of the heat pulses resulting from titration of (A) buffer to eIF4E, (B) eIF4GII_551-745_ to eIF4E and (C) sLb^pro^ to eIF4GII_551-745_/eIF4E complex. The bottom graphs show the integrated heat change in Kcal/mol of injectant. The binding curves were best fitted to a one binding site model (B and C). No change in heat is observed for the addition of buffer (A).

**FIGURE S5.** Selected sLb^pro^C51A mutants, which abrogate ternary complex formation and delay eIF4GII_551-745_ cleavage. Complex formation was analysed via SEC on a HiLoad 16/60 Superdex 200 prep grade column together with aprotinin (6.5 kDa) as an internal standard. 0.5 mg of the complexed proteins were analysed together with 1 mg of aprotinin. Complex formation of proteins was performed at 4°C for 10 minutes. **(A)** eIF4GII_551-745_, eIF4E and sLb^pro^C51A/C133S **(B)** eIF4GII_551-745_, eIF4E and sLb^pro^C51A/Q185R/E186K **(C)** eIF4GII_551-745_, eIF4E and sLb^pro^C51A/C133S/Q185R/E186K. No stable ternary complex between eIF4GII_551-745_, eIF4E and neither mutant sLb^pro^C51A was detected, since two separate peaks (eIF4GII_551-745_/eIF4E and sLb^pro^C51A mutant) could be observed. **(D – F)** *In vitro* cleavage reaction of 5 µM of eIF4GII_551-745_ in the absence or the presence of 5 µM eIF4E by 7 nM of the mutant sLb^pro^: (D) sLb^pro^C51A/C133S, (E) sLb^pro^C51A/Q185R/E186K and (F) sLb^pro^C51A/C133S/Q185R/E186K. Cleavage reactions (total volume 8 µl) were performed at 37°C for the indicated times and the reaction was stopped by the addition of 5x Laemmli sample buffer. Samples were then analysed on a 17.5% SDS PAGE gel and stained with Coomassie blue. Cleavage products are indicated.

**FIGURE S6.** Intensity changes of ^15^N eIF4GII_551-745_ upon titration with wt sLb^pro^C51A or sLb^pro^C51A/C133S/Q185R/E186K to ^15^N eIFGII_551-745_. (A) Residue plots of the titration experiment of ^15^N eIF4GII_551-745_ and sLb^pro^C51A (1:2, grey) and of ^15^N eIF4GII_551-745_ and sLb^pro^C51A/C133S/Q185R/E186K (1:2, red). In the upper panel, the sLb^pro^C51A (grey) is in the foreground, in the lower panel the mutant sLb^pro^C51A/C133S/Q185R/E186K (red) is in the foreground. A native ^15^N HSQC spectrum of eIF4GII_551-745_ was recorded as control before every titration experiment and compared to that obtained after addition of the indicated protein(s). Normalized intensities of every assigned residue were calculated. Residues for which no amide backbone assignment value could be attributed are shown with a value of -0.1. The white boxes represent eIF4E binding site (BS), short black boxes the Lb^pro^ BS and grey boxes represent the picornaviral cleavage sites (CS). (B) Overlaid ^15^N HSQC spectra of ^15^N eIF4GII_551-745_ (blue), ^15^N eIF4GII_551-745_ and sLb^pro^C51A (grey) and ^15^N eIF4GII_551-745_ and sLb^pro^C51A/C133S/Q185R/E186K (red).

**FIGURE S7.**  Residue plot of intra- and intermolecular PREs for ^15^N eIF4GII_551-745_ and ^15^N eIF4GII_551-745_ and sLb^pro^C51A. (A) Paramagnetic spectrum of ^15^N eIFGII_551-745_. The spin label MTSL was attached to C638 on ^15^N eIFGII_551-745_ (black star). Normalized signal intensities were calculated using a diamagnetic control spectrum recorded after spin label reduction (not shown). Significant PREs with low intensity ratios were found for residues 606-715. (B) MTSL was attached to C133 on the unlabelled, inactive single cysteine sLb^pro^ sLb^pro^C51A/C125S/C153S (black star) and the mutant added to ^15^N eIFGII_551-745_. Normalized intensities were calculated using a diamagnetic control spectrum of ^15^N eIFGII_551-745_ with sLb^pro^C51A/C125S/C153S recorded after spin label reduction. Significant PREs with smaller intensity ratios on ^15^N eIFGII_551-745_ were found for the stretches of residues 628-631 (eIF4E binding site) and residues 638-655 (between the eIF4E and Lb^pro^ binding site), indicating regions where C133 from sLb^pro^C51A/C125S/C153S comes into spatial proximity to ^15^N eIFGII_551-745_. Residues for which no amide backbone assignment value could be attributed are shown with a value of -0.1. The white boxes represent eIF4E binding site (BS), short black boxes the Lb^pro^ BS and grey boxes represent the picornaviral cleavage sites (CS).

**FIGURE S8.** Protein sequence alignment based on the three-dimensional structure of HRV2 2A^pro^ ([P04936](http://www.uniprot.org/uniprot/P04936)) with CVB4 2A^pro^ (P08292) using the ClustalW2 online tool ^1^. The residues of the active site are shown in reverse video. The sequence identity is 39%, the similarity 55%.

1. Larkin MA, Blackshields G, Brown NP, Chenna R, McGettigan PA, McWilliam H, Valentin F, Wallace IM, Wilm A, Lopez R and others (2007) Clustal W and Clustal X version 2.0. Bioinformatics 23:2947-2948.

2. Gross JD, Moerke NJ, von der Haar T, Lugovskoy AA, Sachs AB, McCarthy JE, Wagner G (2003) Ribosome loading onto the mRNA cap is driven by conformational coupling between eIF4G and eIF4E. Cell 115:739-750.

3. Gradi A, Foeger N, Strong R, Svitkin YV, Sonenberg N, Skern T, Belsham G (2004) Cleavage of eukaryotic translation initiation factor 4GII within foot-and-mouth disease virus-infected cells: identification of the L-protease cleavage site in vitro. J. Virol. 78:3271-3278.
