# Supplementary figures and images for: NMR analysis of the interaction of picornaviral proteinases Lb and 2A with their substrate eukaryotic initiation factor 4GII
Source: Protein Sci. 2015 Oct 4;24(12):1979–96. doi: 10.1002/pro.2807 (PMC4815241; doi:10.1002/pro.2807)

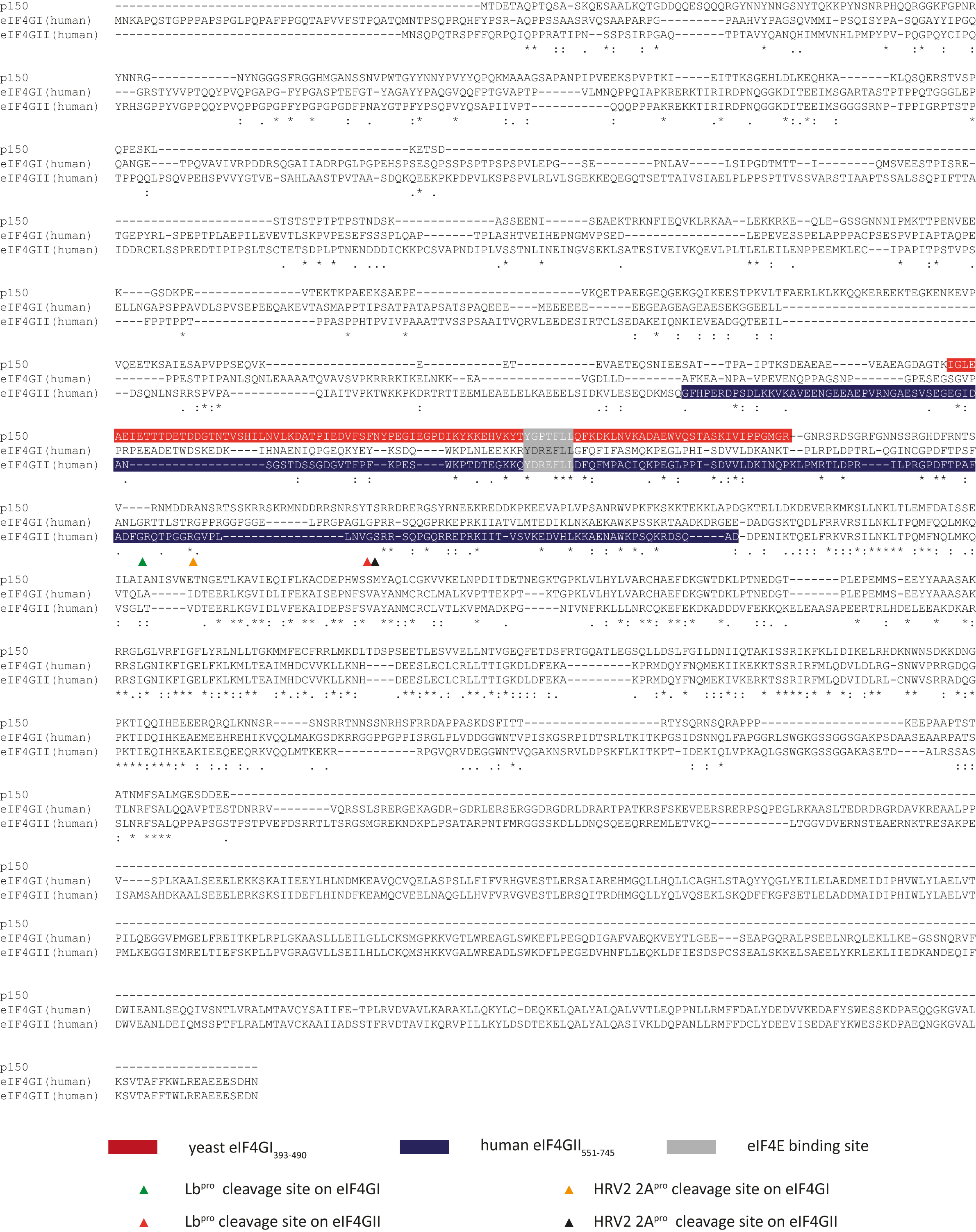

Supplement: Supplementary file 2 — Supporting Information Figure S1 [file PRO-24-1979-s002.tif]

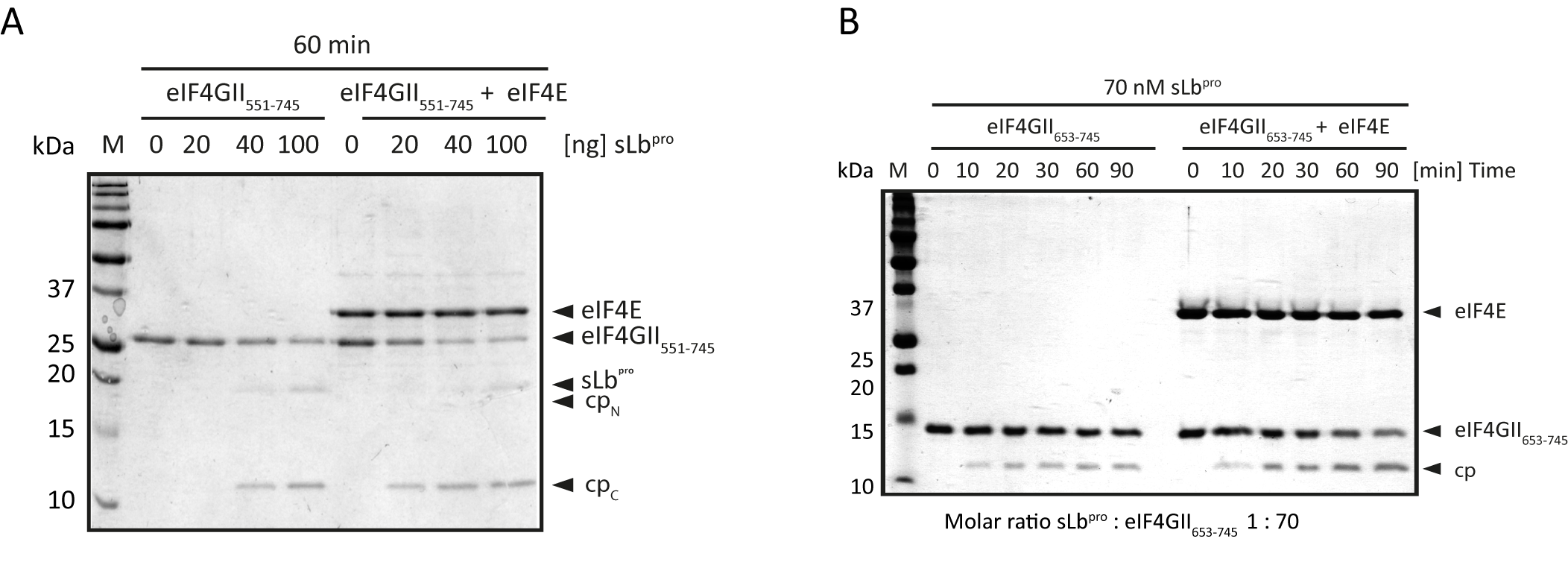

Supplement: Supplementary file 3 — Supporting Information Figure S2 [file PRO-24-1979-s003.tif]

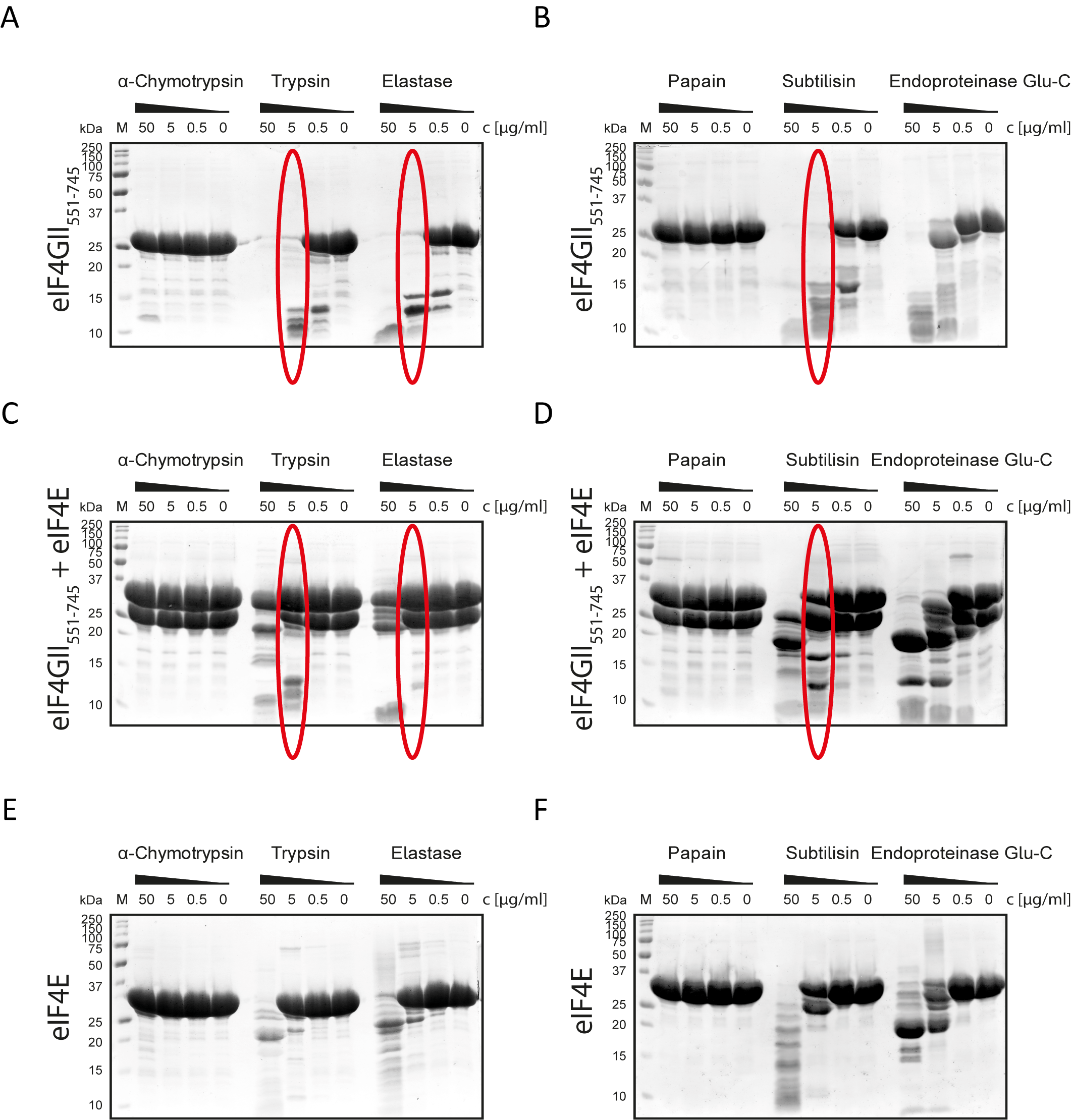

Supplement: Supplementary file 4 — Supporting Information Figure S3 [file PRO-24-1979-s004.tif]

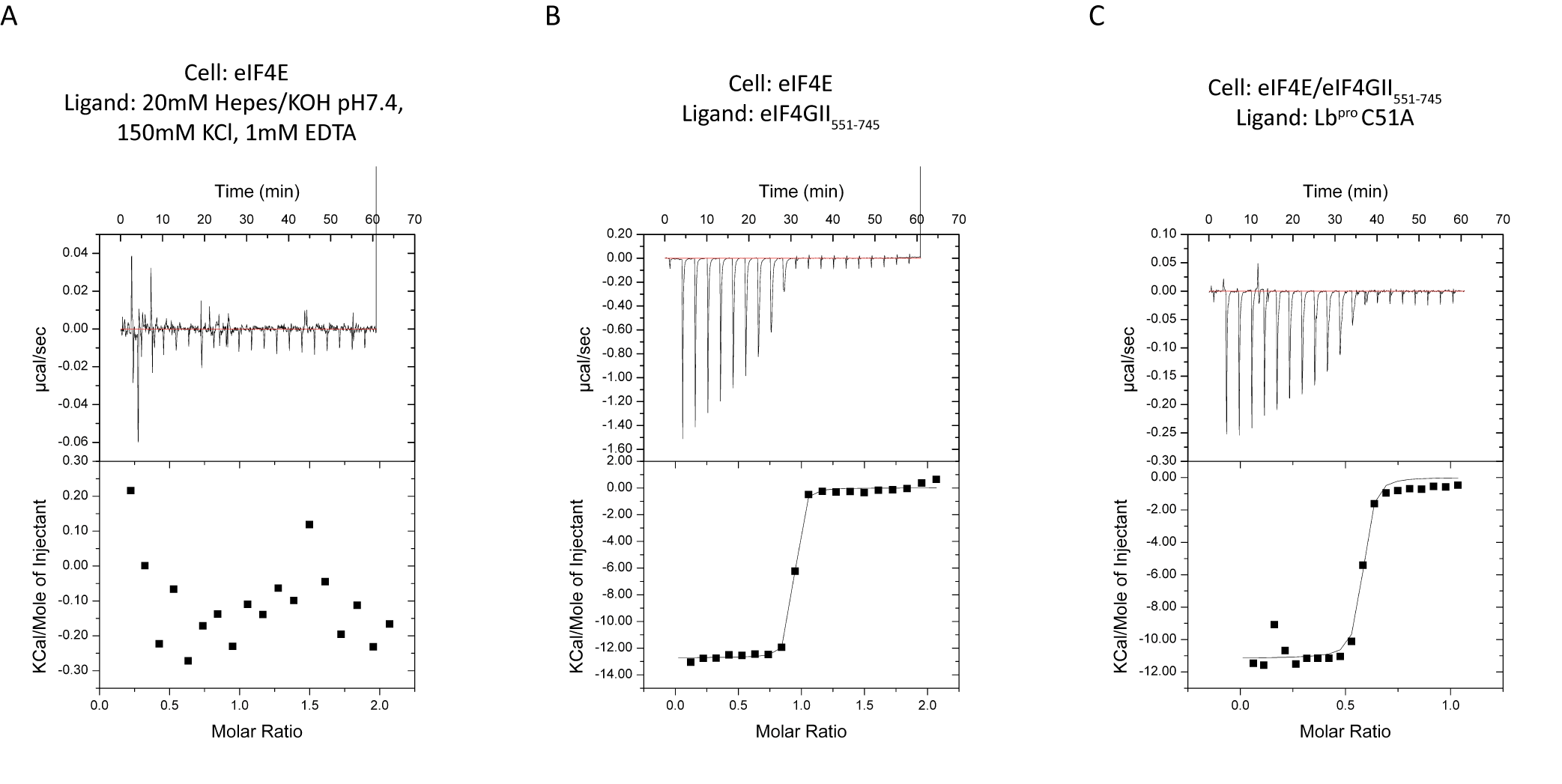

Supplement: Supplementary file 5 — Supporting Information Figure S4 [file PRO-24-1979-s005.tif]

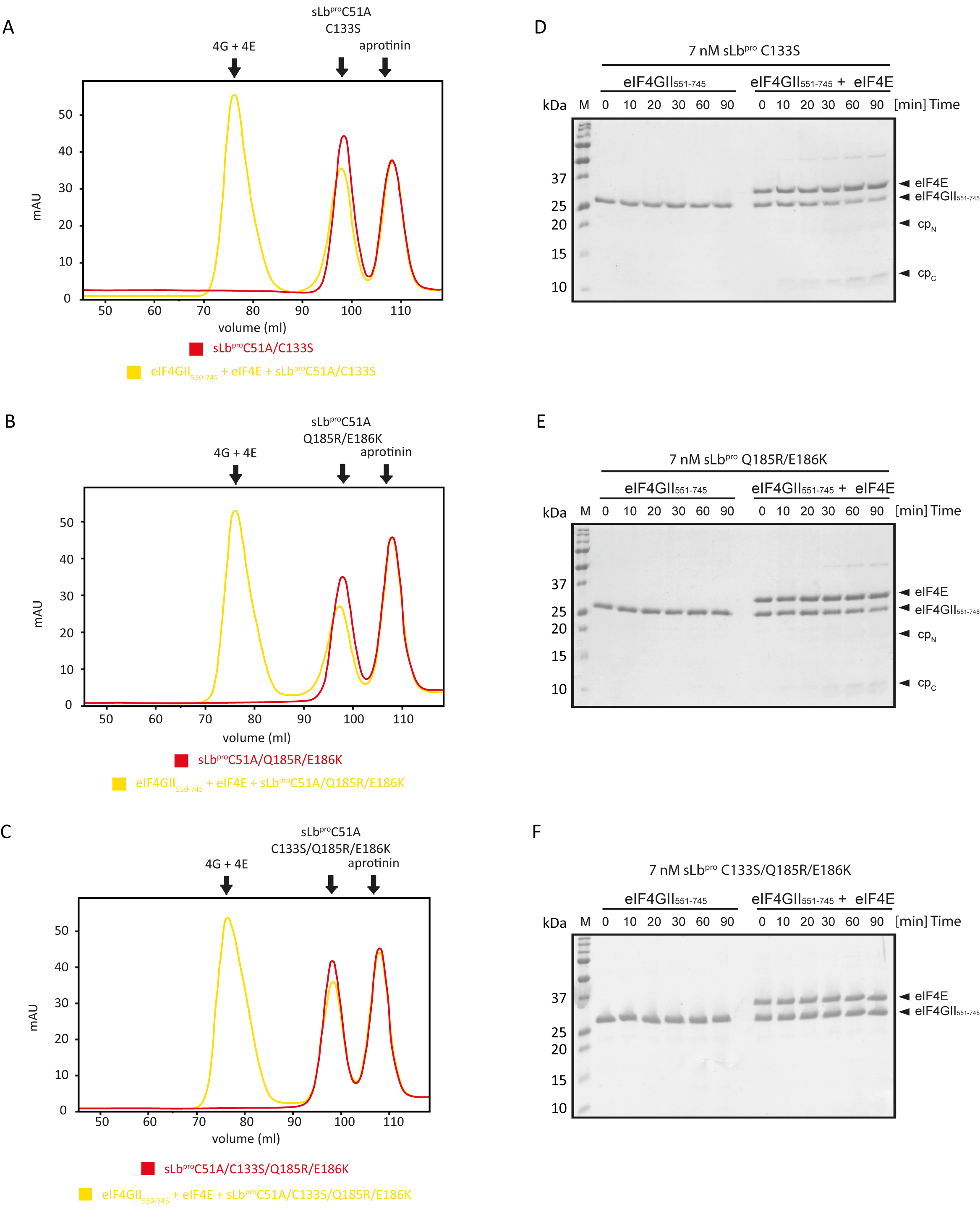

Supplement: Supplementary file 6 — Supporting Information Figure S5 [file PRO-24-1979-s006.tif]

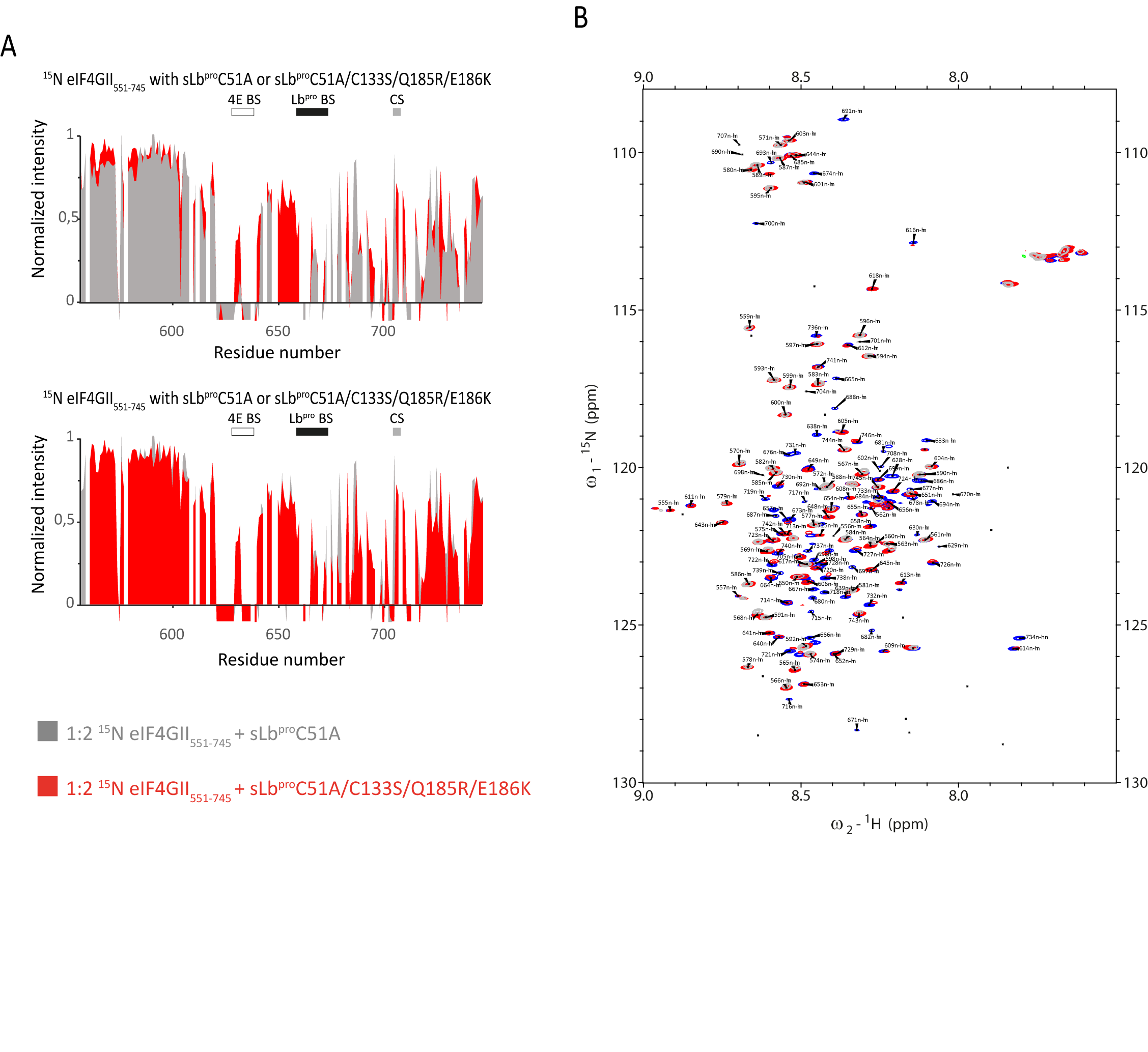

Supplement: Supplementary file 7 — Supporting Information Figure S6 [file PRO-24-1979-s007.tif]

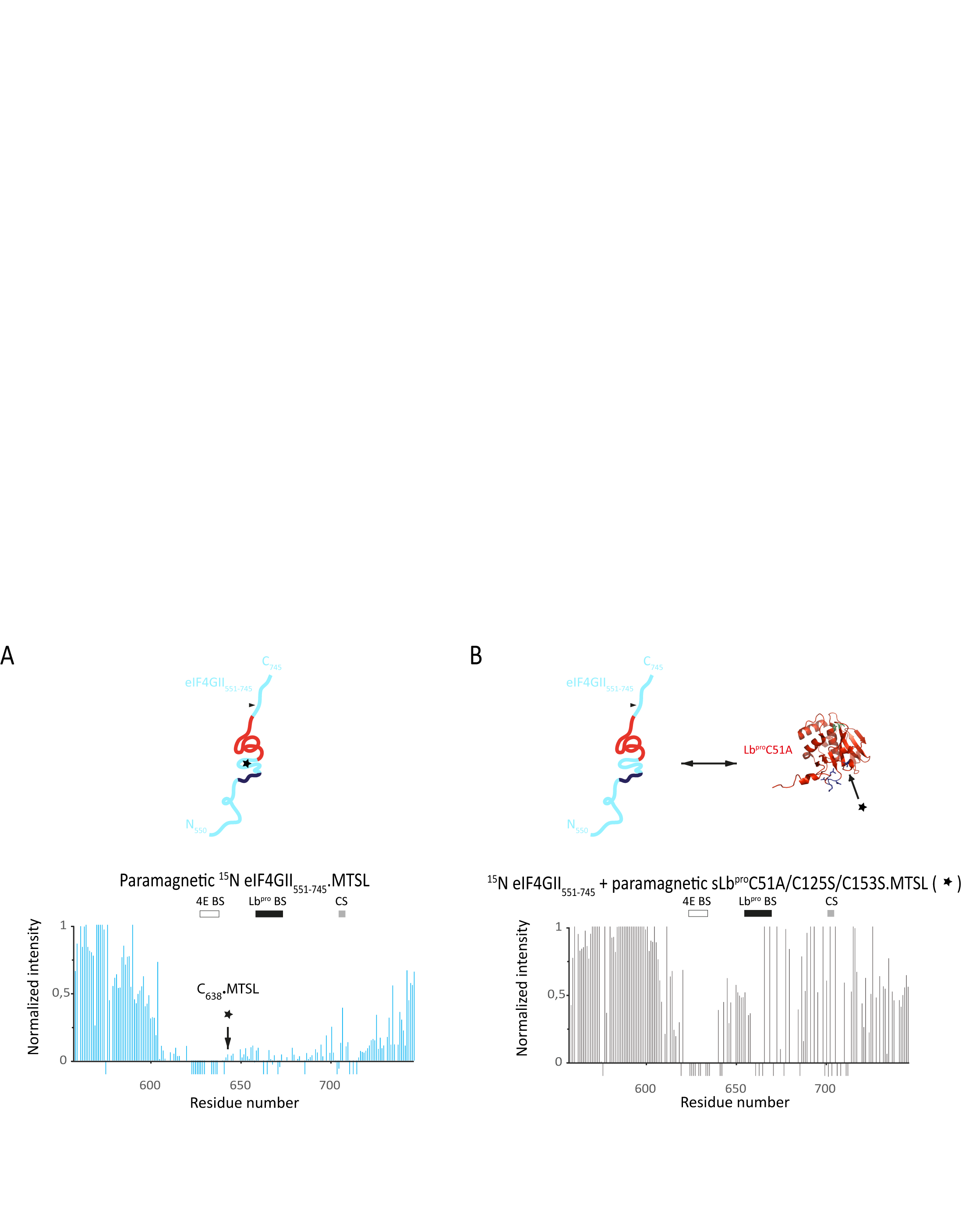

Supplement: Supplementary file 8 — Supporting Information Figure S7 [file PRO-24-1979-s008.tif]

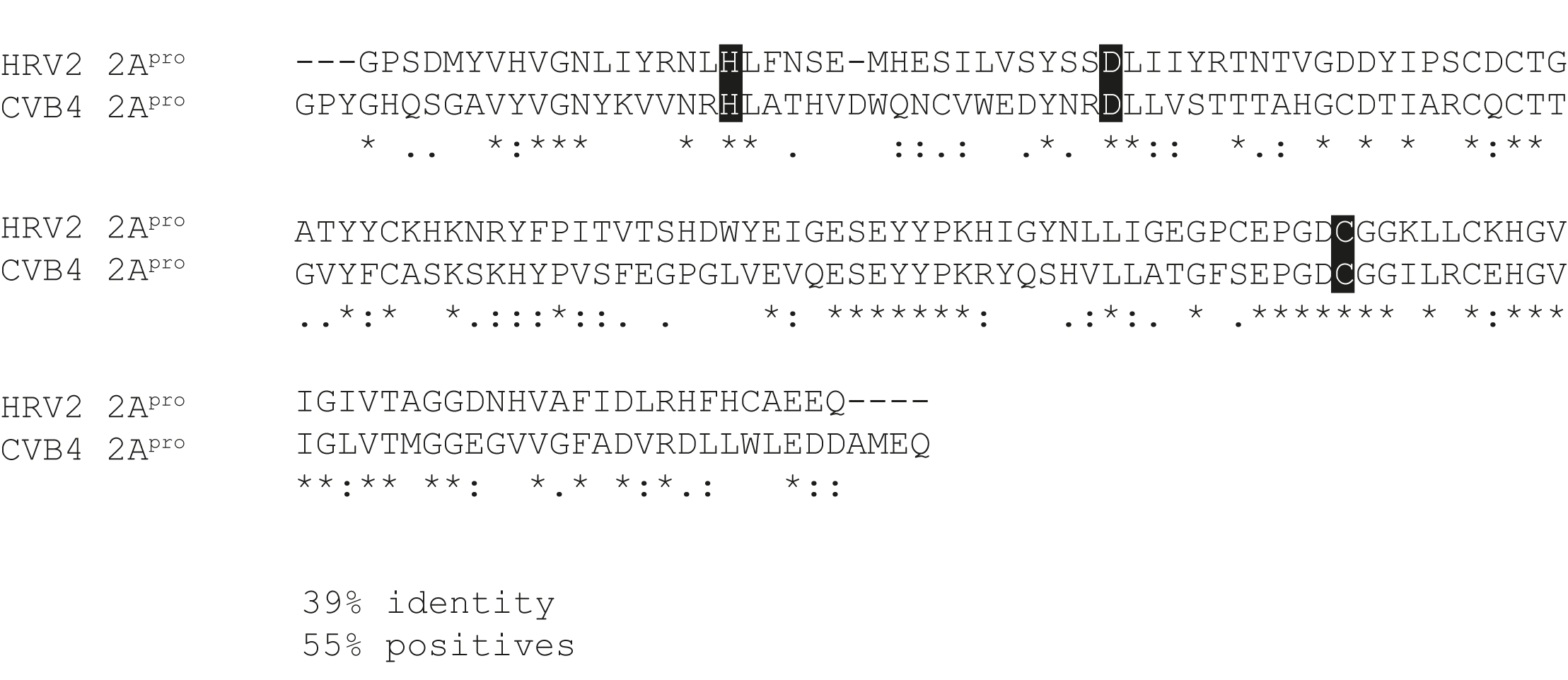

Supplement: Supplementary file 9 — Supporting Information Figure S8 [file PRO-24-1979-s009.tif]
